# Interaction of network and rehabilitation therapy parameters in defining recovery after stroke in a Bilateral Neural Network

**DOI:** 10.1186/s12984-022-01106-3

**Published:** 2022-12-19

**Authors:** Sundari Elango, Amal Jude Ashwin Francis, V. Srinivasa Chakravarthy

**Affiliations:** Computational Neuroscience Laboratory, Department of Biotechnology, Indian Institute of Technology, Madras, India

**Keywords:** Hemiparesis, Rehabilitation, CIMT, BMT, Visuomotor pathway, Computational modelling, Stroke recovery

## Abstract

**Background:**

Restoring movement after hemiparesis caused by stroke is an ongoing challenge in the field of rehabilitation. With several therapies in use, there is no definitive prescription that optimally maps parameters of rehabilitation with patient condition. Recovery gets further complicated once patients enter chronic phase. In this paper, we propose a rehabilitation framework based on computational modeling, capable of mapping patient characteristics to parameters of rehabilitation therapy.

**Method:**

To build such a system, we used a simple convolutional neural network capable of performing bilateral reaching movements in 3D space using stereovision. The network was designed to have bilateral symmetry to reflect the bilaterality of the cerebral hemispheres with the two halves joined by cross-connections. This network was then modified according to 3 chosen patient characteristics—lesion size, stage of recovery (acute or chronic) and structural integrity of cross-connections (analogous to Corpus Callosum). Similarly, 3 parameters were used to define rehabilitation paradigms—movement complexity (Exploratory vs Stereotypic), hand selection mode (move only affected arm, CIMT vs move both arms, BMT), and extent of plasticity (local vs global). For each stroke condition, performance under each setting of the rehabilitation parameters was measured and results were analyzed to find the corresponding optimal rehabilitation protocol.

**Results:**

Upon analysis, we found that regardless of patient characteristics network showed better recovery when high complexity movements were used and no significant difference was found between the two hand selection modes. Contrary to these two parameters, optimal extent of plasticity was influenced by patient characteristics. For acute stroke, global plasticity is preferred only for larger lesions. However, for chronic, plasticity varies with structural integrity of cross-connections. Under high integrity, chronic prefers global plasticity regardless of lesion size, but with low integrity local plasticity is preferred.

**Conclusion:**

Clinically translating the results obtained, optimal recovery may be observed when paretic arm explores the available workspace irrespective of the hand selection mode adopted. However, the extent of plasticity to be used depends on characteristics of the patient mainly stage of stroke and structural integrity. By using systems as developed in this study and modifying rehabilitation paradigms accordingly it is expected post-stroke recovery can be maximized.

**Supplementary Information:**

The online version contains supplementary material available at 10.1186/s12984-022-01106-3.

## Background

Stroke is the second most leading cause of death globally [1] while leaving 50% of the survivors disabled for life [2]. It is caused as a result of loss of blood supply to a part of the brain either due to ischemia (block in a cerebral blood vessel due to a clot) or hemorrhage (rupture of the blood vessel) leading to a lesion. The most common disability after stroke is weakness in the upper limb [3] contralateral to the damaged hemisphere resulting in a condition known as hemiparesis [4].Since the upper limb is compromised, the quality of life after stroke is severely affected [5] and the subjects become dependent on caregivers for their day-to-day lives [6]. In order to restore the lost functionality, physiotherapists often administer physical therapy as a rehabilitative strategy to patients [7].Rehabilitative therapy is of different kinds, depending on the type of setting and movements used (for a detailed review, please see [8]). For example, Constraint-Induced Movement Therapy [9] (CIMT) advocates moving only the affected arm while restraining the intact arm to accomplish various tasks that one might encounter in daily life. This therapy was developed in order to encourage spontaneous use of the affected arm and overcome learned non-use. In contrast, Bimanual Therapy [10] (BMT) endorses moving both arms simultaneously to specific targets placed in the workspace. This therapy was developed in order to provide training for tasks that require bimanual movements, since many tasks in daily life require coordination between the two arms. As can be seen from the definitions, the two therapies given here are apparently contradictory to each other as one views the healthy arm to be opposing the paretic arm while the other views it as being assistive. However, these therapies can be grouped together broadly as task-oriented rehabilitation [11] as they are administered as tasks to be completed by the patients. In such therapies, patients are often advised to keep practicing the task until they are perfected.

Though task-oriented rehabilitation has been around for long, recently, doubts have been raised about its actual efficacy. When using task-oriented rehabilitation practices, it is found that patients are able to perform well only if the testing conditions are identical to the training conditions indicating that it leads to poor retention [12] and less generalization [13]. On the contrary, introducing variability in practice has been shown to increase retention and transfer of skill to other unlearned tasks [14]. Additionally, a new task paradigm devised by Krakauer and Cortés [15] has proposed that encouraging patients to make self-chosen movements in a rich environment can lead to higher recovery levels compared with dull, repetitive task-oriented practice. Further, since the movements are self-chosen, the entire movement/action space is available for the patients to explore and are not restricted by the constraints dictated by a task. Hence, the complexity of the movement is higher under such a paradigm compared to task-oriented rehabilitation. (However, it should be noted that this paradigm was tested on a subset of patients with subacute stroke, with whom it has not yielded encouraging results [16]. Therefore, this technique still needs more vigorous testing, with multiple patient groups, before being accepted as a mainstream rehabilitation technique). Thus, there are several parameters that need to be considered while choosing a rehabilitation paradigm.

In this study, we wanted to develop a computational model capable of understanding the effect of these parameters on recovery after stroke and how they need to be modified under different patient characteristics. We chose reaching as the patient behaviour to replicate with the model. Stroke was then induced in the model, and recovery patterns were observed. Several computational models implement a similar process of capturing the reaching behaviour of humans (for an extensive review, see [17]). One approach taken by these models is to replicate the brain's sensory-motor loop [18–20]. The movement performed is captured by the sensory module and fed back to the motor module, which uses it to compare the difference between intended action and performed action similar to the brain. The model then tries to reduce the error between the intended and actual movements. Models can also be developed for specific rehabilitation therapy to understand its effect on recovery [21]. However, since these models have separate modules dedicated for each brain area, as the complexity of the movement increases, the model's computational cost also increases while still being designed only to address a specific task or a class of tasks or implement a particular therapy. There are also several predictive models that use patient data (lesion size, location, time from onset, initial impairment, etc.) to predict recovery over a time frame [22, 23]. But such models usually only explain recovery in a particular group of patients while classifying the rest as outliers incapable of complete recovery.

For the current study, we wanted to replicate the reaching behaviour observed in stroke patients. Though other models have attempted to do this, they all face the same issue of being very restricted in use only with specific therapies or specific patient data. The aim of our study was to develop a model that can be used with multiple therapies in order to allow a comparison between them to find the optimal therapy for a given set of patient characteristics.

Recently several studies have shown the similarity in activity between the different convolutional layers of the convolutional neural networks (CNNs) and sensory systems in the brain—both visual [24] and auditory cortex [25]. Additionally, these studies have also shown that optimizing the network architecture for a given task, results in connectivity similar to that observed in the brain [25]. Taking this as inspiration, we used convolutional layers on the input side of the network to model the visual side of the visuomotor pathway. The output side of the network consists of a multilayer perceptron, with a single hidden layer, whose output is the muscle activations required to reach a given target in 3D space. The network is trained as a whole with the input being an image consisting of a sphere representing the target position in 3D space while the output is the muscle activations required by the arm to reach the target. The network is organised as a bilateral architecture to resemble the bilateral organisation of the brain. Similar to the two hemispheres of the brain, the CNN is divided into two halves, where the output from each half controls the movement of the respective arm. For easy depiction, we assume that each half of the network controls the corresponding ipsilateral arm, instead of the more realistic contralateral arm. By inducing stroke in one of the halves, we were able to selectively impair the corresponding arm resulting in hemiparesis. To this model of hemiparesis, rehabilitation therapies of different kinds were then administered to and the performance after each was measured and compared. By this comparison, we were able to identify optimal rehabilitation protocol for each condition of stroke. However, it is to be noted that with the current network, we try to map the behaviour of the network to the behaviour seen in stroke patients, in particular their reaching behaviour. Thus, with the current model we are not trying to replicate the neural activity pattern of every layer independently in the cortex, but only model a gross similarity with the visuomotor pathway of the brain, with the lower layers of the network representing the visual areas and the higher layers, the motor areas. Further, in order to reduce the model complexity, we have greatly simplified the working of both the visual and motor areas to only those required for the task at hand.

## Methods

### Arm model

**Fig. 1:**
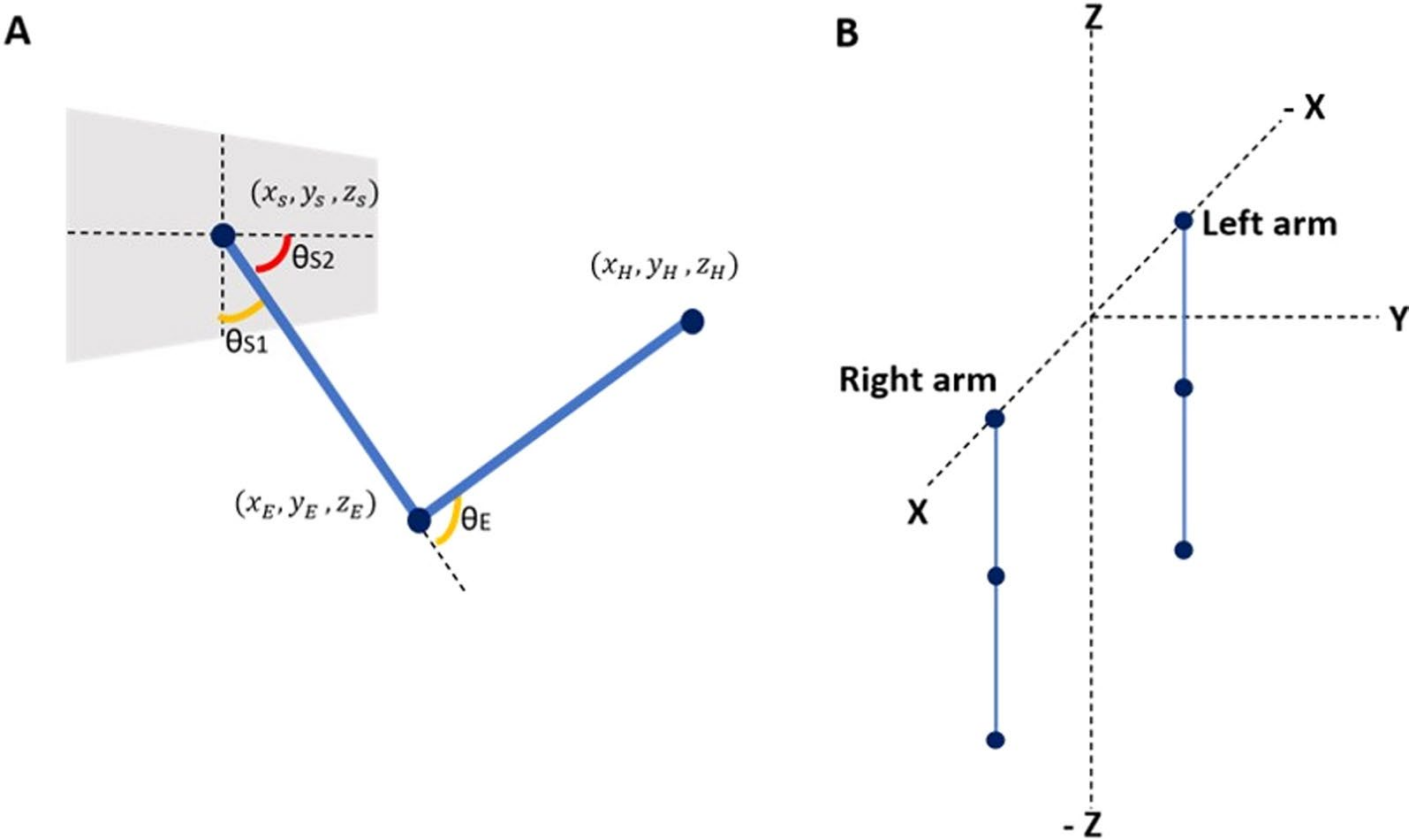
**A** A two link arm model with 2DoFs at the shoulder and 1DoF at the elbow. **B** The configuration of the two arms at initial instant before performing rotations

A two-link arm model is used with three degrees of freedom: (i) elbow flexion, (ii) shoulder flexion, and (iii) shoulder rotation and is hence capable of moving in a 3D workspace. Each degree of freedom is controlled by an agonist–antagonist muscle pair with each arm controlled by 6 muscles in total (schematic shown in Fig. 1). The network controls two such arms—right and left. The output of the network is the 12 muscle activations required for the arms to reach a visually presented target in the 3D-workspace. The origin of the workspace is considered to be in the hollow of the neck, between the clavicle bones of the shoulder. The right shoulder is at coordinates [0.15,0,0] and the left shoulder is at coordinates [− 0.15,0,0]. The muscle activations obtained at the output of the network are used to calculate the elbow and shoulder angles, which is then used to find the position of the end effector. This position is then compared with the actual target position given as input to the network (described in the next section). The error between these two values is then backpropagated through the network, and the weights of the network are modified accordingly.

Angles from the corresponding muscle activations are calculated as follows,

$${\text{Shoulder}}\,{\text{flexion}}:\,{\uptheta }_{{{\text{S}}1}} \, = \,\left( {{\text{MN}}_{{{\text{Ag}}\left( {S1} \right)}} - {\text{MN}}_{{{\text{An}}\left( {{\text{S}}1} \right)}} } \right)\frac{{\uppi }}{2} + \frac{{\uppi }}{2}$$

$${\text{Shoulder}}\,{\text{rotation}}:{\uptheta }_{{{\text{S}}2}} \, = \,\left( {{\text{MN}}_{{{\text{Ag}}\left( {S2} \right)}} - {\text{MN}}_{{{\text{An}}\left( {{\text{S}}2} \right)}} } \right)\frac{{\uppi }}{2} + \frac{{\uppi }}{2}$$

$${\text{Elbow}}\,{\text{flexion:}}\,{\uptheta }_{{\text{E}}} = \left( {{\text{MN}}_{{{\text{Ag}}\left( E \right)}} - {\text{MN}}_{{{\text{An}}\left( {\text{E}} \right)}} } \right)\frac{{\uppi }}{2} + \frac{{\uppi }}{2}$$

where, ${\mathrm{MN}}_{\mathrm{Ag}}$ is the muscle activation input to the agonist muscle and ${\mathrm{MN}}_{\mathrm{An}}$ is the muscle activation to the antagonist muscle.

$${\text{Rot}}_{{\text{E}}}^{{\text{R}}} = \left[ {\begin{array}{*{20}c} {\cos {\uptheta }_{{\text{E}}} } & 0 & { - \sin {\uptheta }_{{\text{E}}} } \\ 0 & 1 & 0 \\ {\sin {\uptheta }_{{\text{E}}} } & 0 & {\cos {\uptheta }_{{\text{E}}} } \\ \end{array} } \right]$$

$${\text{Rot}}_{{{\text{S}}1}}^{{\text{R}}} = \left[ {\begin{array}{*{20}c} {\cos {\uptheta }_{{{\text{S}}1}} } & 0 & { - \sin {\uptheta }_{{{\text{S}}1}} } \\ 0 & 1 & 0 \\ {\sin {\uptheta }_{{{\text{S}}1}} } & 0 & {\cos {\uptheta }_{{{\text{S}}1}} } \\ \end{array} } \right]$$

$${\text{Rot}}_{{{\text{S}}2}}^{{\text{R}}} = \left[ {\begin{array}{*{20}c} {\cos {\uptheta }_{{{\text{S}}2}} } & { - \sin {\uptheta }_{{{\text{S}}2}} } & 0 \\ {\sin {\uptheta }_{{{\text{S}}2}} } & {\cos {\uptheta }_{{{\text{S}}2}} } & 0 \\ 0 & 0 & 1 \\ \end{array} } \right]$$

$${\text{Rot}}_{{\text{E}}}^{{\text{L}}} = \left[ {\begin{array}{*{20}c} {\cos {\uptheta }_{{\text{E}}} } & 0 & {\sin {\uptheta }_{{\text{E}}} } \\ 0 & 1 & 0 \\ { - \sin {\uptheta }_{{\text{E}}} } & 0 & {\cos {\uptheta }_{{\text{E}}} } \\ \end{array} } \right]$$

$${\text{Rot}}_{{{\text{S}}1}}^{{\text{L}}} = \left[ {\begin{array}{*{20}c} {\cos {\uptheta }_{{{\text{S}}1}} } & 0 & {\sin {\uptheta }_{{{\text{S}}1}} } \\ 0 & 1 & 0 \\ { - \sin {\uptheta }_{{{\text{S}}1}} } & 0 & {\cos {\uptheta }_{{{\text{S}}1}} } \\ \end{array} } \right]$$

$${\text{Rot}}_{{{\text{S}}2}}^{{\text{L}}} = \left[ {\begin{array}{*{20}c} {\cos {\uptheta }_{{{\text{S}}2}} } & {\sin {\uptheta }_{{{\text{S}}2}} } & 0 \\ { - \sin {\uptheta }_{{{\text{S}}2}} } & {\cos {\uptheta }_{{{\text{S}}2}} } & 0 \\ 0 & 0 & 1 \\ \end{array} } \right]$$

The above rotation matrices are used to calculate the position of the end effector along with the angles calculate above, as follows,

$${\text{End effector position}}:{\text{H }}_{e}^{{{\text{R}}/{\text{L}}}} = \left[ {\left( {{\text{H}}_{{\text{o}}} - E_{{\text{o}}} } \right)\left( {{\text{ Rot}}_{{\text{E}}}^{{{\text{R}}/{\text{L}}}} } \right) + E_{o} } \right]\left[ {{\text{Rot}}_{{{\text{S}}1}}^{{{\text{R}}/{\text{L}}}} } \right]\left[ {{\text{Rot}}_{{{\text{S}}2}}^{{{\text{R}}/{\text{L}}}} } \right] + S_{o}$$

where, ${\mathrm{H}}_{\mathrm{o}}$, ${\mathrm{E}}_{\mathrm{o}}$ and ${\mathrm{S}}_{\mathrm{o}}$ are the starting coordinates of the hand ([0.15, 0, 0.6] for right and [− 0.15, 0, 0.6] for left (0.6 is the assumed length of the whole arm)), elbow ([0.15, 0, 0.3] for right and [− 0.15, 0, 0.3] for left (0.3 is the assumed length of the upper arm)) and shoulder ([0.15, 0, 0] for right and [− 0.15, 0, 0] for left) respectively. We assumed the arm length to be 0.6 units. The length from the shoulder to elbow and elbow to wrist were assumed to be equal (0.3 units each) to make the computation simpler. We also assumed the shoulder length to be 0.3. The origin is supposed to be centered at the forehead, and hence the left shoulder’s x-coordinate was at − 0.15 while the right was at + 0.15.

### Visual Input Generation

**Fig. 2:**
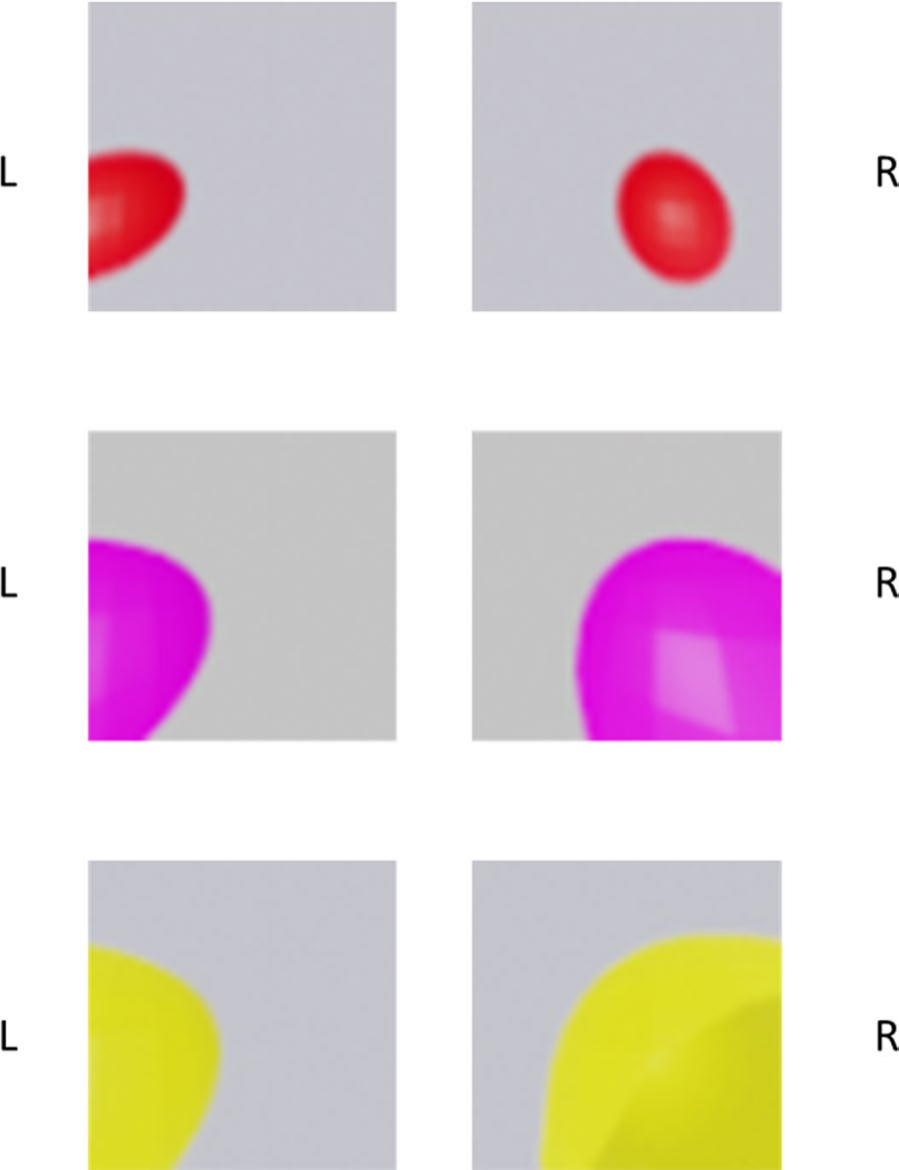
Visual input used in the study

The inputs to the CNN are generated using Blender 2.8 [26, 27], a free and open-source software used to develop animations and video games. For this study, we used the software to create input images for the network. The image consists of a coloured sphere placed at the target position (i.e., centre of the sphere corresponds to the target position), in front of a blank wall. Three such datasets are generated for each position with each dataset differing in colour and size of the sphere to ensure the network learns to reach the centre of the sphere and is not distracted by other features of the object like size or colour. As the position of the target is moved, the size of the sphere increases or decreases depending on its distance from the camera. A light source is used in the Blender environment to illuminate the scene (constraint of the software). Depending on the position of the target with respect to the light source, the sphere casts a shadow of varying size and length. Additionally, a glare is cast on the sphere depending on its position from the light source. In order to ensure that the network does not get biased to one of these features and instead uses them efficiently to estimate the position of the target, we created different datasets with the same target positions, but using spheres of different sizes and colour. Since the target position to be estimated is in 3D, stereovision is incorporated in the input by using two input channels that are laterally shifted (left–right disparity) instead of a single channel. To do this, two cameras are placed in the scene at a distance analogous to the interocular distance. Representative images of the input used are given in Fig. 2.

Along with the images, two binary Hand Selection Parameters (HSPs) are fed to the network that specify which arm is to be moved to reach the target for that particular input. Thus, the hand selection mode is set with the help of HSPs (described in Table 1) and play a vital role in replicating unimanual or bimanual therapy conditions in the network.

**Table 1:** Description of hand selection parameters

HSP#1	HSP#2	One object in the workspace	Two objects in the workspace
0	0	Both arms at their respective home positions	Both arms at their respective home positions
0	1	Right arm moves to target; Left arm at left home position (Unimanual Right Task)	Right arm moves to target in right workspace; Other target is ignored; Left arm at left home position (Unimanual Right Task)
1	0	Left arm moves to target; Right arm at right home position (Unimanual Left Task)	Left arm moves to target in left workspace; Other target is ignored; Right arm at right home position (Unimanual Left Task)
1	1	Both arms move to the same target (Bimanual Joint Task)	Each arm moves to the corresponding target (Bimanual Independent Task)

Rest* position for Left Arm—(0.15,0.27,0.3); Right Arm—(0.15,0.27,0.3). (*Calculated by setting activations for all muscles at 0.5)

As we can see, from the table, four types of movement are considered—no movement, bimanual, unimanual right and unimanual left. Under each condition, the network is trained with 3 different coloured targets of 3 sizes at 1174 target positions (total—4*3*1174). For testing, 450 target positions were considered (total—4*3*450).

### Testing the performance of the network

For the optimization of the network architecture, performance of the network over all targets and all actions (specified by the two HSPs) is tested. Quantification of the performance is expressed in terms of Reaching Error (RE) defined as follows,

$$RE = \left\| {X_{targ} - X_{arm} } \right\|_{2}$$

where, ${X}_{targ}$ is the 3D coordinates of the target; ${X}_{arm}$ is the 3D coordinates of the arm (obtained from $\mathrm{H}{ }_{e}^{\mathrm{R}/\mathrm{L}}$).

The smaller the value of RE, the higher the performance of the network. For the stroke studies, only the tasks in which either one or both the arms are active are considered i.e., the tasks with the HSPs set to [0 0] are removed from the analysis. This was done because the performance of the arm under rest condition is not affected by stroke and hence measuring recovery under this condition seems meaningless.

### Network architecture

In order to simulate hemiparesis, a network with bilateral symmetry is required. For this, we started with a regular CNN with 5 convolutional layers and 3 fully connected layers, split vertically into two symmetrical halves with each half controlling one arm. The two retinal images of the target are then fed to this network and the output of the network acts as the motor neuronal pool controlling the activations of the muscles of each arm.

**Fig. 3:**
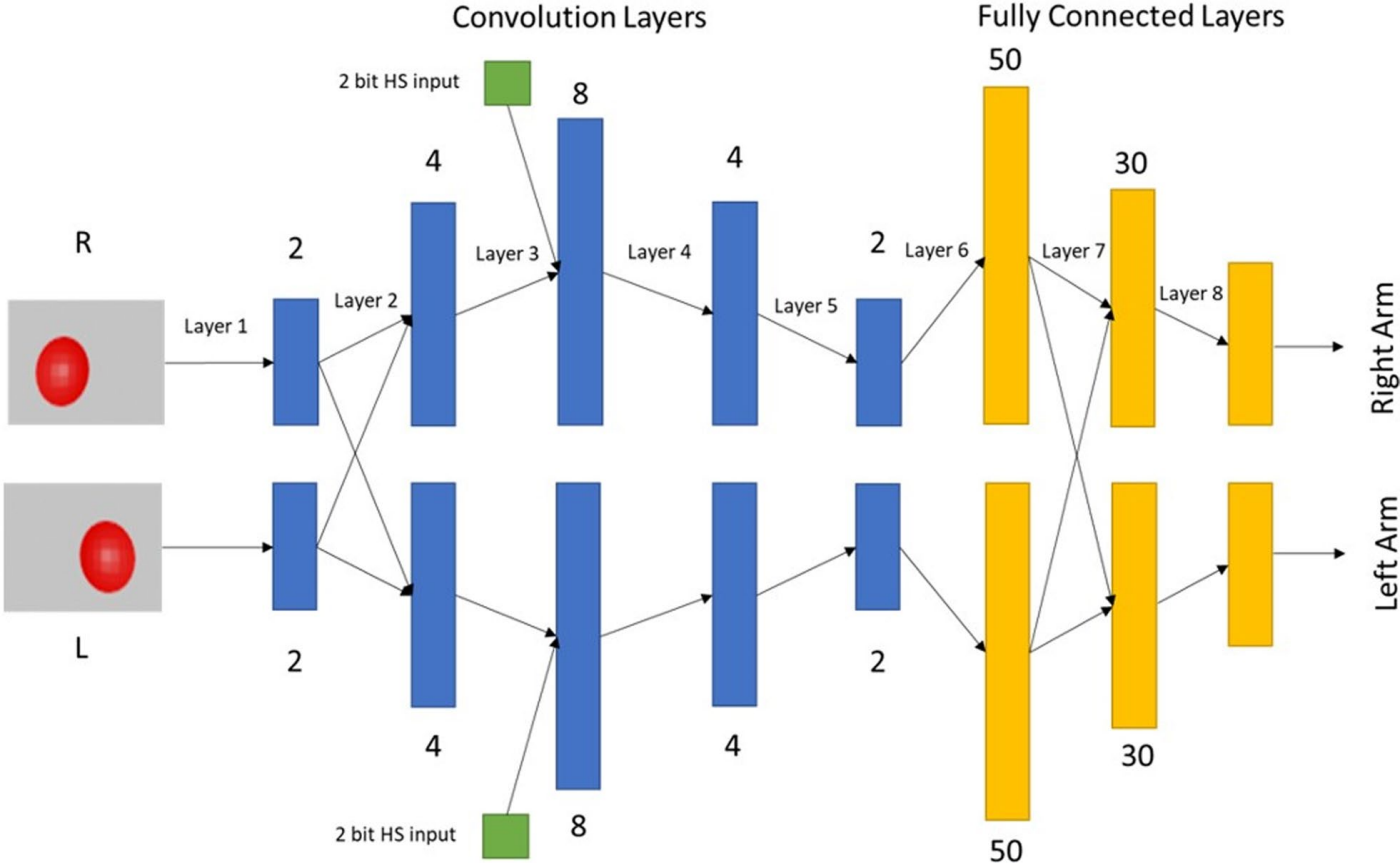
Network architecture used in the study. Blue bars indicate convolutional layers while yellow bars indicate fully connected layers

Since the network is split into two, two kinds of feedforward connections are possible between successive layers of the network—ipsilateral and contralateral. The ipsilateral connections on either side were maintained at 100%—regular connectivity from the previous layer to the next layer at all layers as in a standard CNN. In contrast, the contralateral connections (cross-connections) were optimized and given only at two layers—between the first two convolutional layers and the first two fully connected layers. This network was then used to carry out further studies. The optimization process that led to this architecture is discussed in the Additional File 1 provided (Additional File 1). The optimized architecture is shown below in Fig. 3. The hyperparameters used in the network are listed in Table 2.

**Table 2:** Hyperparameters used in the network

Layer no. and type	Activation function used	Regularization used	No. of feature maps/nodes (L + R)	Kernel Size	Stride length	Pooling size	Pooling Stride length
1 (Convolutional Layer)	Relu	L2	2 + 2	(5,5)	(1,1)	(2,2)	(2,2)
2 (Convolutional Layer)	Relu	L2	4 + 4	(5,5)	(1,1)	–	–
3 (Convolutional Layer)	Relu	L2	8 + 8	(5,5)	(1,1)	–	–
4 (Convolutional Layer)	Relu	L2	4 + 4	(5,5)	(1,1)	–	–
5 (Convolutional Layer)	Relu	L2	2 + 2	(5,5)	(1,1)	–	–
6 (Fully connected layer)	Sigmoid	L2	50 + 50	–	–	–	–
7 (Fully connected layer)	Sigmoid	L2	30 + 30	–	–	–	–
8(Fully connected layer)	Sigmoid	L2	6 + 6	–	–	–	–

Learning rate used with the network is 0.0001

### Lesion study

In this study, two stages of stroke conditions were analyzed—acute and chronic stroke. Stroke is induced by setting the activity of certain selected neurons to zero. After this, various rehabilitation therapies were administered to the network. Recovery pattern after rehabilitation was analyzed in order to understand preference of one therapy over the other under the given stroke conditions.

#### Inducing stroke in the network

##### Stage of recovery: acute stroke

For acute stroke, a few nodes (n = 5, 10, 15 and 20 out of the 30 nodes present) were selected on the left half of the penultimate layer (the layer prior to the last/output layer) of the network and their activities were set to zeros. The lesion was maintained in the same site for all the models in order to ensure uniformity in the resulting impairment. In a clinical setting, this would translate to recruiting patients for a study with similar lesion characteristics. The nodes to be set to zero was selected in order, for e.g., for lesion size of 5 nodes, the nodes 1 to 5 were set to 0, while for a lesion size of 10 nodes, the nodes 1 to 10 were set to 0 and so on. Since the left half of the network was only connected to the left arm, if the lesion was on the left half, only the performance of the left arm was affected. The reduction in performance was reflected in the RE of the left arm measured across targets, while the right arm remained intact and unaffected. In this fashion, hemiparesis was induced in the network. In this study, we only consider the left arm to be the paretic arm, while the right arm is maintained as the healthy arm. Due to the symmetry of the network, the methods/results obtained here for the left arm can be easily translated to the right arm.

##### Stage of recovery: chronic stroke

**Fig. 4:**
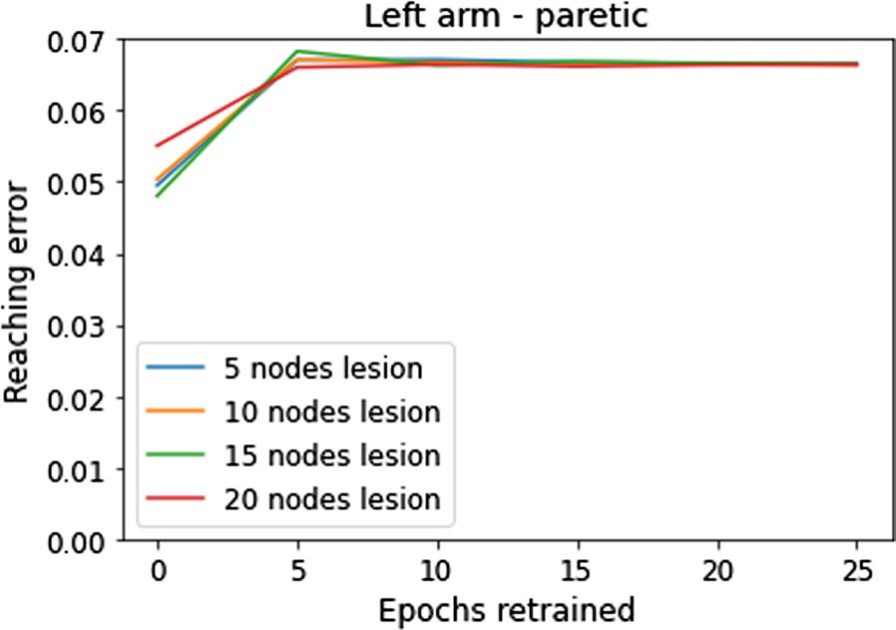
Reaching Error vs Number of epochs retrained for models with different lesion sizes. As the number of epochs used is increased the damage to the network also increases. However, there is a saturation after 10 epochs with the models showing no significant difference in damage with increasing number of epochs

To replicate the learned non-use scenario [28] observed in chronic stroke patients, after inducing the lesion, we trained the model for 10 epochs under HSP = [0 1] modality, thereby encouraging the movement of only the non-paretic arm while the paretic arm is always at rest—at home position (The number of epochs was fixed at 10 as not much increase in damage was observed upon increasing the number of epochs (refer to Fig. 4).). A model trained in such a way is then rehabilitated under the chosen therapeutic protocols, similar to the acute stroke models, and the recovery between the two models is compared. In the current study, a very simplistic arm model is chosen, wherein the properties of the musculature are largely ignored and only the kinematics of the arm are used for modelling. Hence the deterioration of muscles that occur due to and after stroke are not considered while modelling chronic stroke.

The number of epochs used to obtain the chronic model is in line with those used in other similar studies [29] (healthy model uses 30 epochs—yielding a ratio of 1/3 for chronic/healthy similar to a ratio of 1/4 in the Ballester et al. model).

#### Varying corpus callosum (CC) integrity

Reduction in the structural integrity of CC has been observed in some patients after stroke [30–32]. The cross-connections in the network are similar to the CC in the brain. By varying the connection strength of the cross-connections, the structural integrity of the network can be compromised. The cross-connections associated with the penultimate layer was used for this paradigm. To do this, we used a method similar to the one used to create lesions is employed. Thus, for a given percentage of integrity (percentages considered, p = 0.5, 0.7 and 0.9), the corresponding number of cross-connections ((1 − p) × total number of connections) are chosen randomly and these values are set to zero. This is only done to the cross-connections on the motor region of the final optimized network as it is the CC on the motor side that is found to play a greater role in recovery in clinical study [30]. This is done to both acute and chronic stroke models and performance of such a model after rehabilitation was analysed.

#### Administering rehabilitation therapy in the network

##### Analysing effect of complexity of movement: stereotypic vs exploratory movement condition

**Fig. 5:**
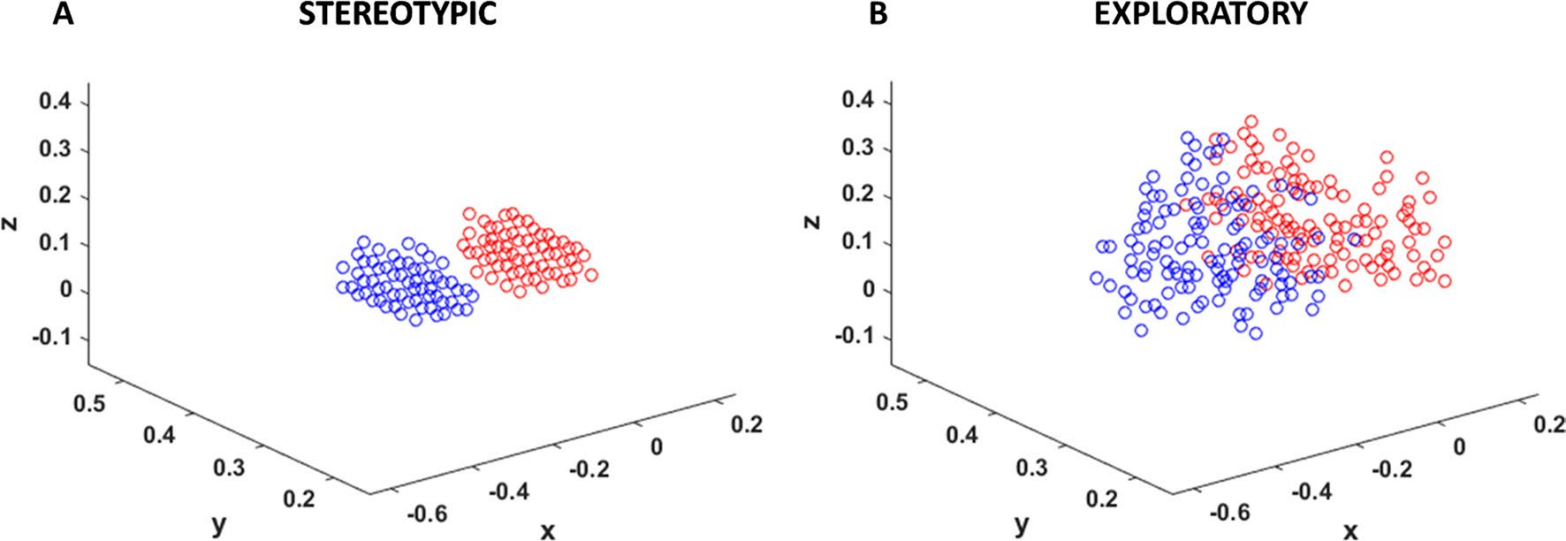
Stereotypic and Exploratory conditions used for therapy/retraining in the model. Red dots indicate targets for right arm, while blue dots indicate targets for left arm

Therapy is administered in two different formats in the network—using stereotypic or exploratory movements. Stereotypic movements are used to replicate task-oriented rehabilitation in which, the movements are inspired by those used in daily life and are restricted to a particular region in the workspace. To capture this, under stereotypic condition, the inputs used are confined to a sphere in the workspace (Fig. 5a). The M points chosen from the sphere are repeatedly presented (N times) during therapy to capture repetitive practice used in task-oriented rehabilitation, resulting in M × N (= P = 300) points used for therapy. For the Exploratory workspace (Fig. 5b), the entire region used during training is used for the rehabilitation, but with much fewer points considered (300 points for rehab vs 1173 for training healthy model). Thus, the final number of points used under both conditions are equal (P = 300 for both). The workspace for Exploratory is defined as follows, for left − 0.6 < x < 0, 0 < y < 0.6, − 0.1 < z < 0.5 and for right—0 < x < 0.6, 0 < y < 0.6, − 0.1 < z < 0.5. Comparatively, the Stereotypic workspace is much smaller, for left—-0.3 < x < − 0.2, 0.25 < y < 0.35, 0.05 < z < 0.15 and for right—0.1 < x < 0.2, 0.25 < y < 0.35, 0.05 < z < 0.15.

##### Analysing effect of hand selection mode: CIMT vs BMT

In order to replicate CIMT, the inputs generated under the Stereotypic and Exploratory conditions are used with the HSPs set to [1 0]. Thus, only the affected arm (left) is moved while the unaffected arm (right) remains at rest (home) position. For BMT, both arms are active, and hence the HSPs are set to [1 1]. Depending on the number of targets (objects) in the workspace, the arms either reach the same target or reach separate targets under BMT condition.

##### Analysing the effect of the extent of plasticity: global vs local

For patients with high impairment or in the chronic phase of stroke, when conventional therapy fails, studies have shown that integrating multisensory stimuli in the environment can help in recovery [33]. This is made possible because they often involve activation of multiple sensory areas which serve as alternative pathways that can be used by the brain to compensate for the cell loss caused by the stroke. Trying to capture the essence of this philosophy, we varied the number (or strength) of connections that were available for retraining. Under local plasticity condition, only the connections associated with the lesioned layer were retrained. Whereas under global plasticity condition, the connections in the entire network were retrained.

### Statistical analysis

The mean RE across the input space was the final parameter used to measure performance. For the stroke studies, 5 models trained with random weight initialization were used and the mean values averaged across these 5 individually trained models were used for final analysis and comparison. Since there are many treatment groups to be compared, one-way ANOVA test was used followed by a Tukey HSD test. The significance level was set at *p*_*sig*_ = 0.05. Before running the ANOVA test, Levene test was used to check for homogeneity of variance. For those groups/conditions that violated this test, Welch correction was applied to the ANOVA test and Dunn-Bonferroni test was selected as the post-hoc test for these groups. Again, significance value was set at *p*_*sig*_ = 0.05. All the analyses were run on Python implementation using appropriate modules/libraries. The p-values obtained for each comparison is provided in Additional File 2.

## Results

In this study, we present a computational model of bimanual reaching which can be used to map stroke characteristics to parameters of rehabilitation. To achieve this, we used a CNN model to replicate the visuomotor pathway in the brain. The characteristics of stroke chosen were lesion size, time since stroke onset (Acute vs Chronic) and structural integrity of the network. Similarly, three parameters were chosen to define the rehabilitation paradigm—complexity of movement, participation of the unaffected arm during rehabilitation (movement type) and the number of connections available (local vs global) for retraining.

### Acute stroke

**Fig. 6:**
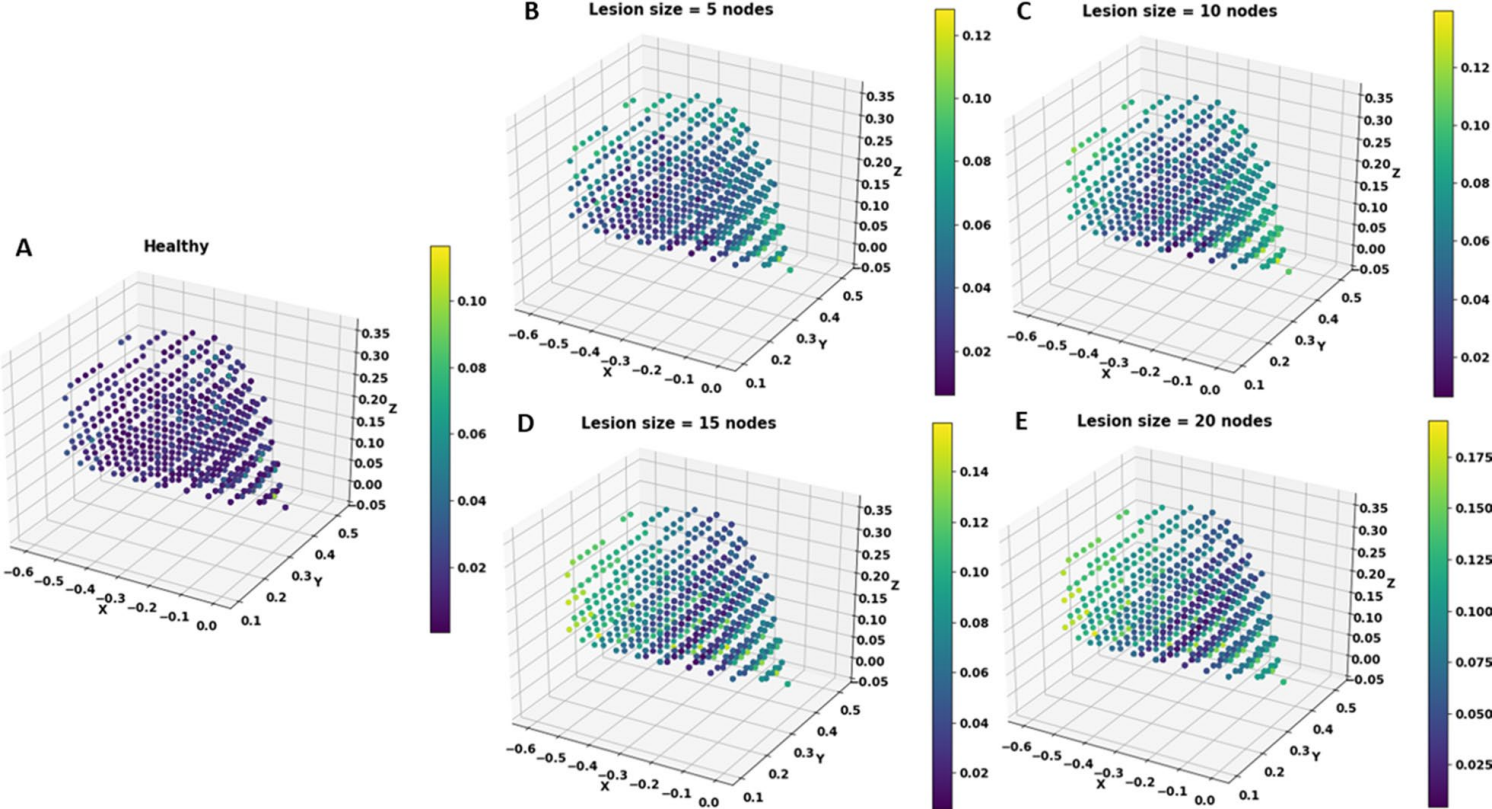
Reaching Error across workspace for **A** Healthy network and stroke-induced network with a lesion size of **B** 5, **C** 10, **D** 15 and **E** 20 nodes. Lesion is induced in the penultimate layer of the network which has a total of 30 nodes. As the lesion size increases, the number of targets showing error increases along with the value of the error (indicated by the color bar given to the right side of each plot)

**Fig. 7:**
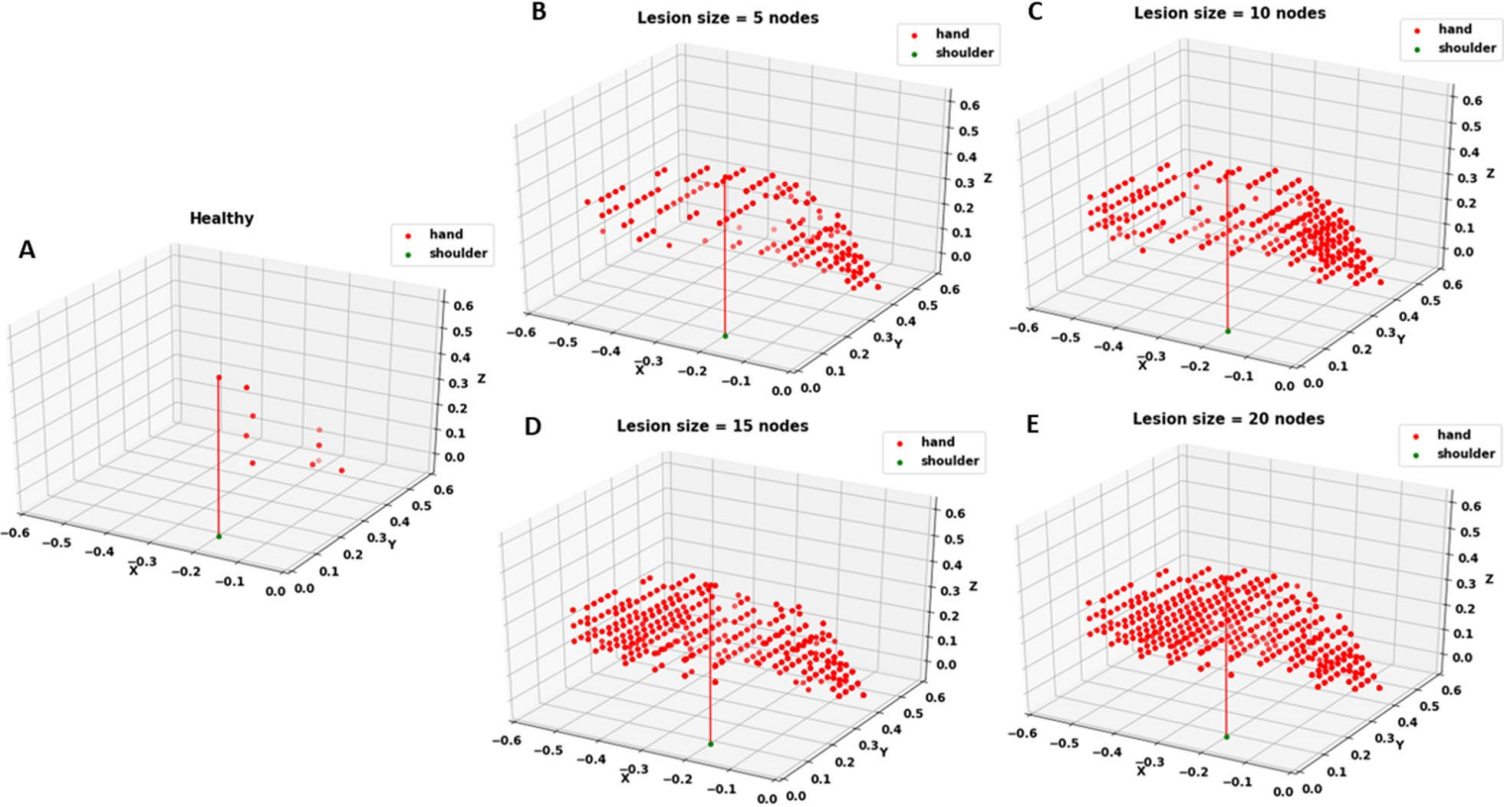
Targets showing RE > 0.6 for **A** Healthy network and stroke-induced network with a lesion size of **B** 5, **C** 10, **D** 15 and **E** 20 nodes. Lesion is induced in the penultimate layer of the network which has a total of 30 nodes. As the lesion size increases, the number of targets crossing the error threshold increases. The red line indicates the starting position of the arm. It is provided to show that error starts increasing from the edges of the workspace

**Fig. 8:**
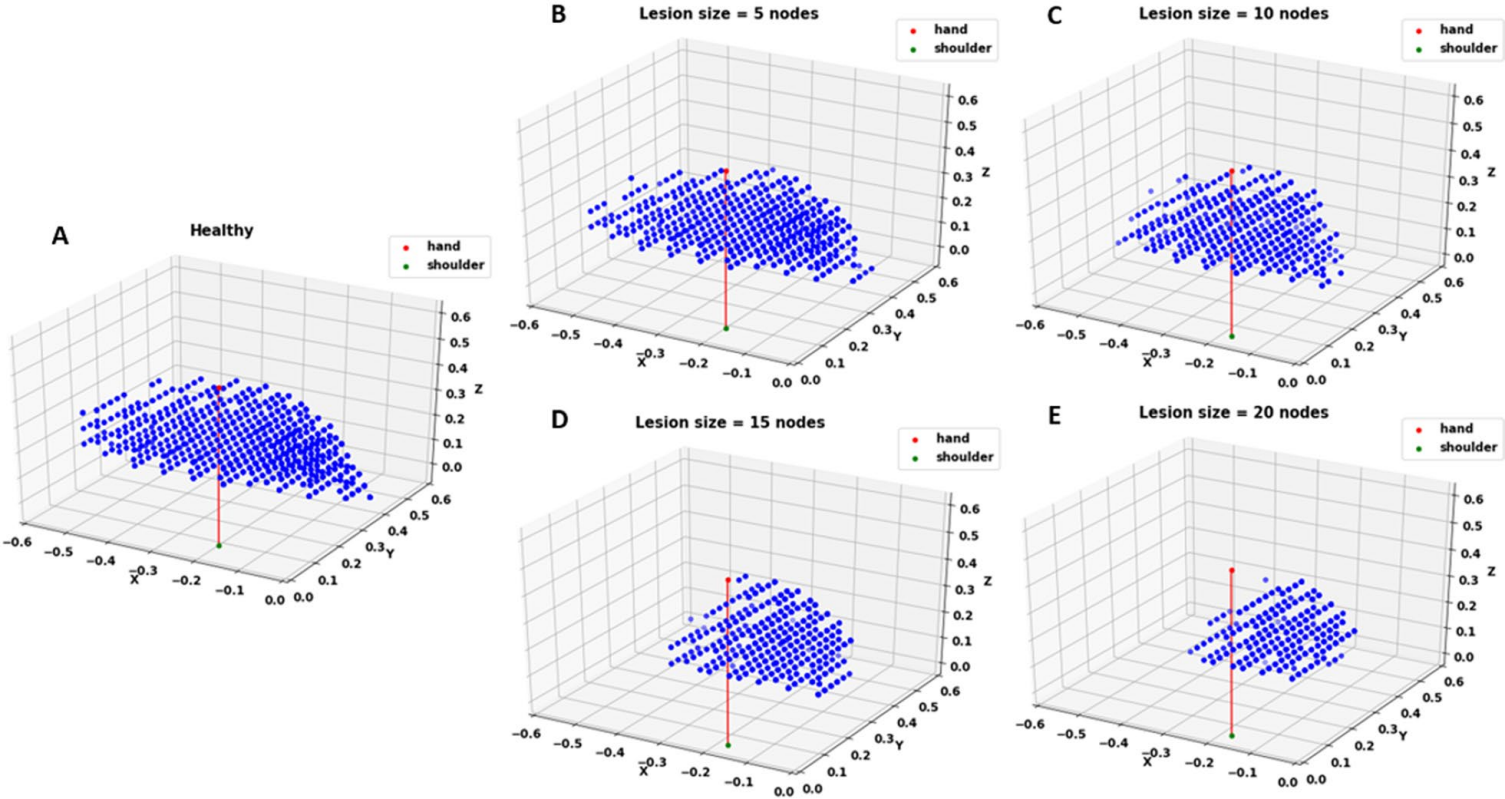
Targets showing RE < 0.6 for **A** Healthy network and stroke-induced network with a lesion size of **B** 5, **C** 10, **D** 15 and **E** 20 nodes. Lesion is induced in the penultimate layer of the network which has a total of 30 nodes. As the lesion size increases, the number of targets below the error threshold decreases. The red line indicates the starting position of the arm. It is provided to show that as the lesion size increases, the workspace gets restricted to areas closer to the arm

Acute stroke was induced in the network by setting the activity of a few selected nodes to zeros. This in turn reduces the performance of the network which can be quantified by the Reaching Error (RE) defined as the Euclidean distance between the desired (target) and the actual position of the arm. The plots shown in Fig. 6 indicate the error exhibited by the network before (healthy case) and after inducing lesion. From the plot for the healthy network, it can be seen that the network is able to reach to almost all the points in the workspace efficiently. However, a network with a lesion finds it difficult to reach certain areas and this inaccessible area seems to expand as the lesion size is increased. By inaccessible area, we mean targets with RE greater than a threshold of 0.06 (this value is chosen since it is 1/10th the actual length of the arm). In the Fig. 6, we have plotted the error (RE) shown by the network for a given target. Each of the points in the 3D plot represents a target position presented as input to the network. The colour of the point represents the reaching error (colour code varies for each lesion size, as the maximum error shown by the network for each lesion size varies. Colour bar depicting the colour code used is given next to each of the plots). From the figure, we can see that as the lesion size increases, the network has increasing number of targets with high error (colour of the targets becomes more yellow, and less blue). This is more explicitly shown in Fig. 7. which exhibits only those targets for which the RE of the network crosses the threshold. For a healthy network, this consists of very few points and only of points in the edges of the workspace. Whereas for a stroke affected network, as lesion size increases, more and more of the workspace is filled with such points. Figure 8. represents the inverse of Fig. 7. i.e., it shows the number of targets having RE lesser than 0.06. In this figure, we see that the number of targets keep decreasing as lesion size increases, thus indicating that the network’s ability to reach to targets in its workspace efficiently keeps decreasing with increasing lesion size. Thus, we conclude that the available workspace for the network also reduces. With the stroke induced in the network, the network’s ability to attempt to move to a target does not change, only it’s efficiency. If the RE crosses 0.06, we conclude that it was a poor attempt, hence the corresponding target is unreachable for the network.

#### Effect of complexity of movement used for therapy: stereotypic vs exploratory

**Fig. 9:**
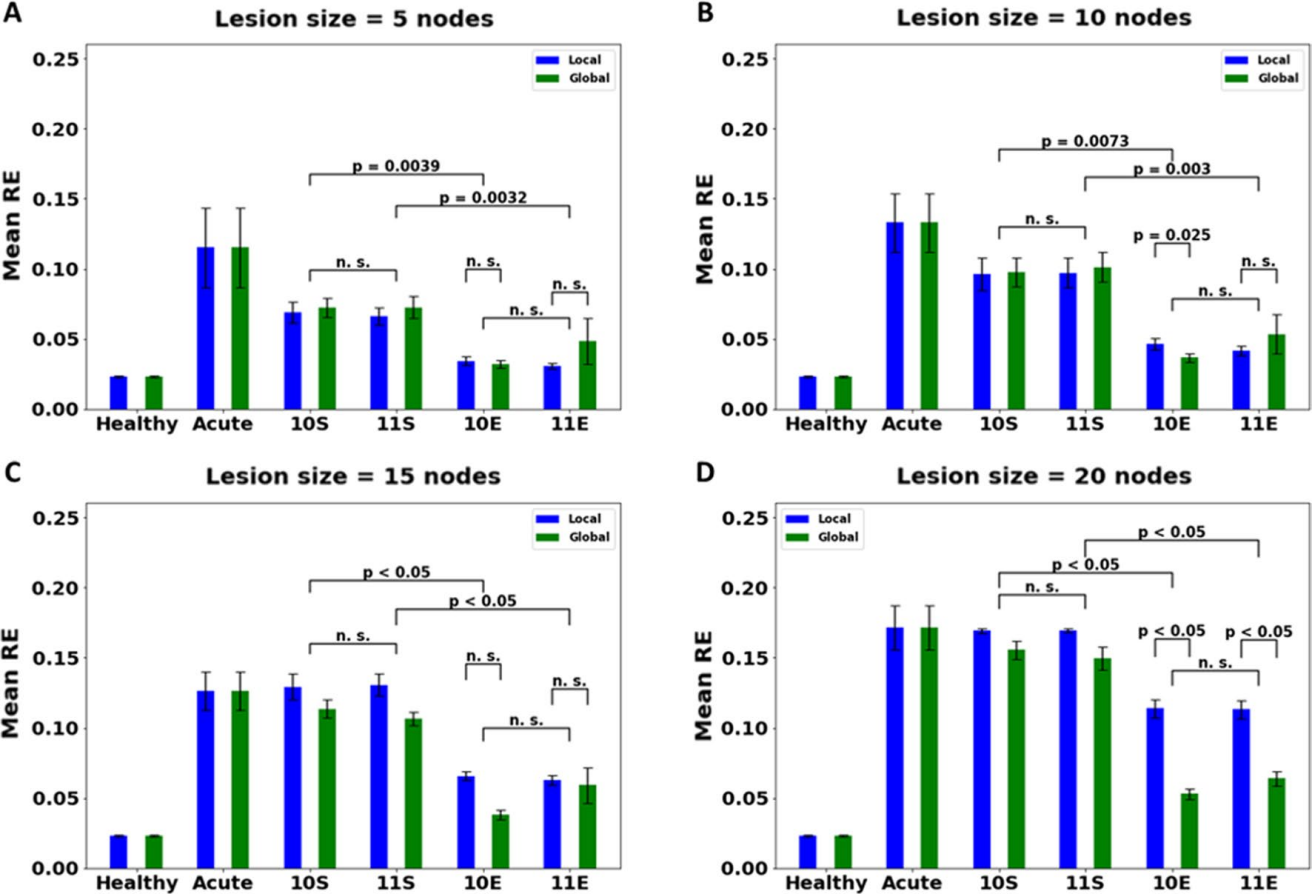
Comparison of different therapies under global and local plasticity conditions for acute stroke model with a lesion size of **A** 5, **B** 10, **C** 15 and **D** 20 nodes. Lesion is introduced in the penultimate layer of the network consisting of 30 nodes. [Key for x-axis: 10S (CIMT Stereotypic), 11S (BMT Stereotypic), 10E (CIMT Exploratory), 11E (BMT Exploratory)]. For both hand movement conditions, exploratory therapy works better than the corresponding stereotypic therapy across all lesion sizes (p < 0.05 upon comparing mean RE for network after therapy with 11S vs 11E and 10S vs 10E, and p > 0.1 when comparing therapies within E therapy i.e., 10E vs 11E and within S therapy i.e., 10S vs 11S). Upon comparing the performance based on plasticity condition, we can see that no significant changes are observed when the size of the lesion is small (p > 0.1 (n.s.) for therapy 11E under local vs global plasticity condition and for therapy 10E under local vs global plasticity condition. S therapies were not compared for plasticity condition since we see the E therapy is better than S). However, for larger lesions, global plasticity condition is better (p < 0.05 for lesion size = 20 nodes (Fig. 8D) for therapy 11E under local vs global plasticity condition and for therapy 10E under local vs global plasticity condition). With higher lesion size, the resulting damage is also higher. Hence the network requires more connections in order to recover

For rehabilitation, we chose movements with two different levels of complexity—Stereotypic and Exploratory. Under stereotypic condition, the inputs chosen for rehabilitation belonged to a small area confined by a sphere in the workspace while for exploratory condition, no such restriction was placed and the entire workspace was open for “exploring”. As the entire workspace is available under the exploratory condition, the complexity of the movement used increases. This way, we were able to analyse the effect of complexity of movement used on recovery. From the plots shown below (Fig. 9), we see that the performance of the network after rehabilitation with exploratory movements is better than the performance after rehabilitation with stereotypic movements (p < 0.05 for all lesion sizes, while comparing corresponding exploratory therapy with stereotypic therapy) regardless of the hand selection mode (CIMT or BMT). This effect is consistent across lesion sizes and for smaller lesion sizes (n = 5, 10), rehabilitation with exploratory workspace brings the network's performance to levels comparable to a healthy network (Fig. 9). Since this result is achieved regardless of the type of the movement used, we can conclude that the dependence of recovery of the network is on the type of movement complexity and not the choice of hand selection (moving only the affected arm vs. moving both arms). Also, as the lesion size increases, performance does not seem to improve upon using a stereotypic workspace for rehabilitation and the error remains at the same level after rehabilitation as it was before (Fig. 9).

#### Effect of hand selection mode used for therapy: CIMT vs BMT

To check the effect of movement of the unaffected arm on the performance of the affected arm, we selected two types of movements—move only the affected arm (CIMT) and move the affected along with the unaffected arm (BMT) during therapy. Upon comparing the performance of the network after these two movements as rehabilitation therapies (Fig. 9), no visible trend emerges (p > 0.1—not significant (n.s.)). Both hand selection modes seem to improve the performance of the network equally and in both therapies, using exploratory movements improves performance better than using stereotypic movements. This is true across all lesion conditions and under both conditions of plasticity. This result stresses again the importance of the complexity of movements used to improve the network's performance rather than the hand selection mode used during rehabilitation.

#### The effect of the number of connections retrained: local vs global plasticity

In order to check the effect of the number of connections available for retraining, we used two conditions—global and local. When the network was trained under global plasticity condition, connections across the entire network are available for retraining. However, under local plasticity, only the afferent connections associated with the lesioned layer are retrained. Comparing the performance of the network trained with global plasticity with local plasticity, it is found that for smaller lesion sizes (n = 5, 10, 15), there is not much difference between the two cases (*p* > 0.2) (Fig. 9). However, for the extreme case of lesion size = 20, performance improves only upon using global plasticity. But here again, a significant difference is not found between using CIMT or BMT but using exploratory movements does prove more beneficial compared to stereotypic movements.

### Chronic stroke

**Fig. 10:**
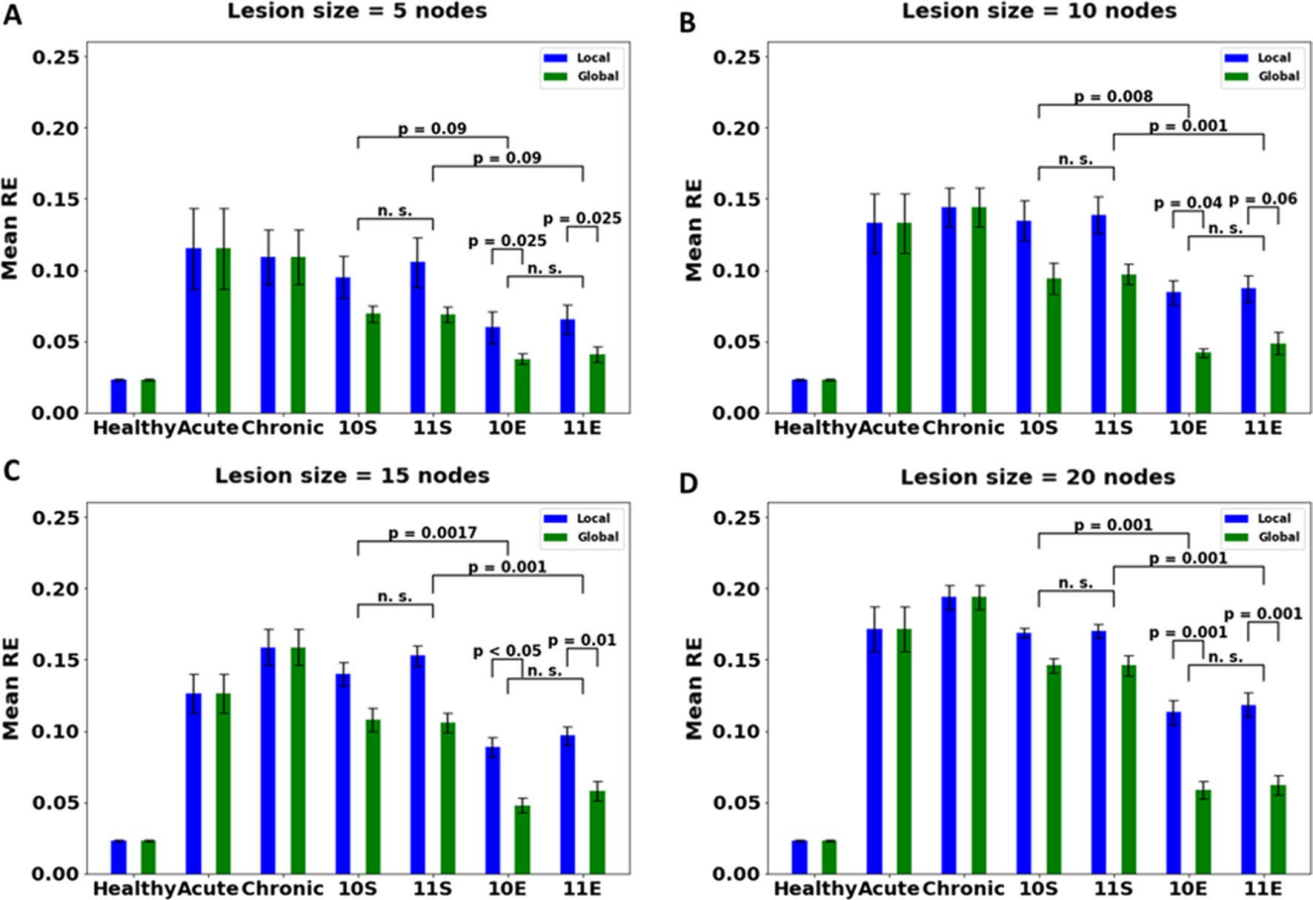
Comparison of different therapies under global and local plasticity conditions for chronic stroke model with lesion size of **A** 5, **B** 10, **C** 15, and **D** 20 nodes. Lesion is induced in the penultimate layer containing a total of 30 nodes. [Key for x-axis: Chronic Stroke (C), 10S (CIMT Stereotypic), 11S (BMT Stereotypic), 10E (CIMT Exploratory), 11E (BMT Exploratory)]. Similar to acute stroke models, chronic stroke models also show a preference to exploratory movements over stereotypic (p < 0.05 upon comparing mean RE for network after therapy with 11S vs 11E and 10S vs 10E, and p > 0.1 when comparing therapies within E therapy i.e., 10E vs 11E and within S therapy i.e., 10S vs 11S). However, under chronic condition, the network prefers global plasticity over local plasticity even for smaller lesion sizes (p < 0.05 for all lesion size for therapy 11E under local vs global plasticity condition and for therapy 10E under local vs global plasticity condition). This shows that, for the same lesion size, damage in the network is higher in the chronic condition than in the acute condition, hence needing more connections for recovery

Chronic stroke is induced in the network after training the network with hand selection mode set to [0 1] modality for 10 epochs after the lesion has been induced. The plots given in Fig. 10 show performance under this condition. Due to the repeated use of the non-paretic arm, the paretic arm’s performance deteriorates further.

#### Effect of hand selection mode used for therapy: CIMT vs BMT

The network is retrained under CIMT and BMT with both stereotypic and exploratory movements similar to the acute stroke models. As observed in the acute stroke models, the performance of the chronic stroke model is significantly higher under exploratory movements than stereotypic movements with both CIMT and BMT (Fig. 10). Again, similar to the acute stroke model, no significant difference is found between retraining with CIMT and BMT.

#### Effect of plasticity

On training under global plasticity condition, the network performance (Fig. 10) improves significantly better than under local plasticity for all lesion sizes regardless of therapy used. Unlike acute models, chronic stroke models do not show a lesion size-specific selectivity for global plasticity.

### Effect of integrity of CC on recovery

With the acute and chronic models discussed above, we were able to establish that using an exploratory movement condition was better than using stereotypic movement condition. Hence, for recovery after loss of CC integrity, only CIMT and BMT under exploratory condition were considered. The two different plasticity conditions—global and local—were still used. Optimal plasticity condition for rehabilitation was found for each of the models.

#### The effect of CC integrity on acute stroke

**Fig. 11:**
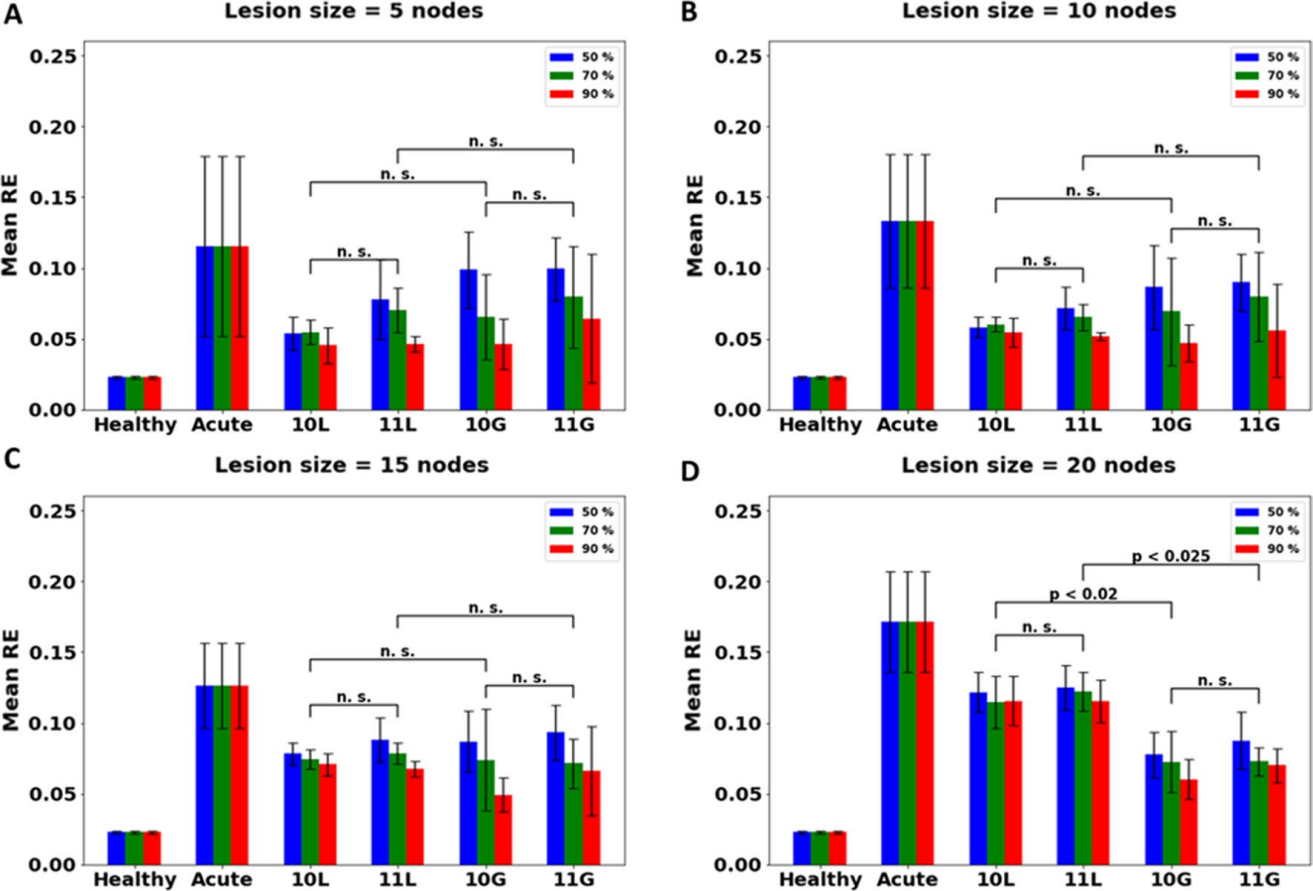
Comparison of different therapies for acute stroke model with lesion size of **A** 5, **B** 10, **C** 15 and **D** 20 nodes, under different values of structural integrity of CC are given. Lesion is induced in the penultimate layer having a total of 30 nodes. [Key for x-axis: 10L (CIMT Exploratory under local plasticity), 11L (BMT Exploratory under local plasticity), 10G (CIMT Exploratory under global plasticity), 11G (BMT Exploratory under global plasticity)]. When the lesion size is low (5, 10 and 15 nodes), the network shows no preference for either plasticity condition over all values of structural integrity of the CC (p > 0.1 when comparing 10L with 10G and 11L with 11G. Each therapy is compared with the other under the same integrity condition). However, for lesion size of 20, global is preferred over local plasticity (p < 0.02 when comparing 10L with 10G and p < 0.025 when comparing 11L with 11G). These results are similar to what is observed in acute stroke condition with 100% integrity (Fig. 8)

Under acute stroke, after reducing the integrity of the CC, the network is trained under CIMT and BMT with an exploratory workspace under local and global plasticity condition. As the integrity of the CC is reduced, the recovery achieved under all therapies reduces. For all values of CC integrity, the network seems to prefer global plasticity to local plasticity for lesion size n = 20 (Fig. 11D). Thus, for a lesion size of 20, for all integrity values (including 100%) it is recommended to go for global plasticity condition. Whereas for all other conditions of lesion sizes and integrity values, no significant difference is found between the two plasticity conditions, and hence, local plasticity is recommended (since global is not significantly better than local, and global is more computationally expensive, local is recommended over global, for the network).

#### The effect of CC integrity on chronic stroke

**Fig. 12:**
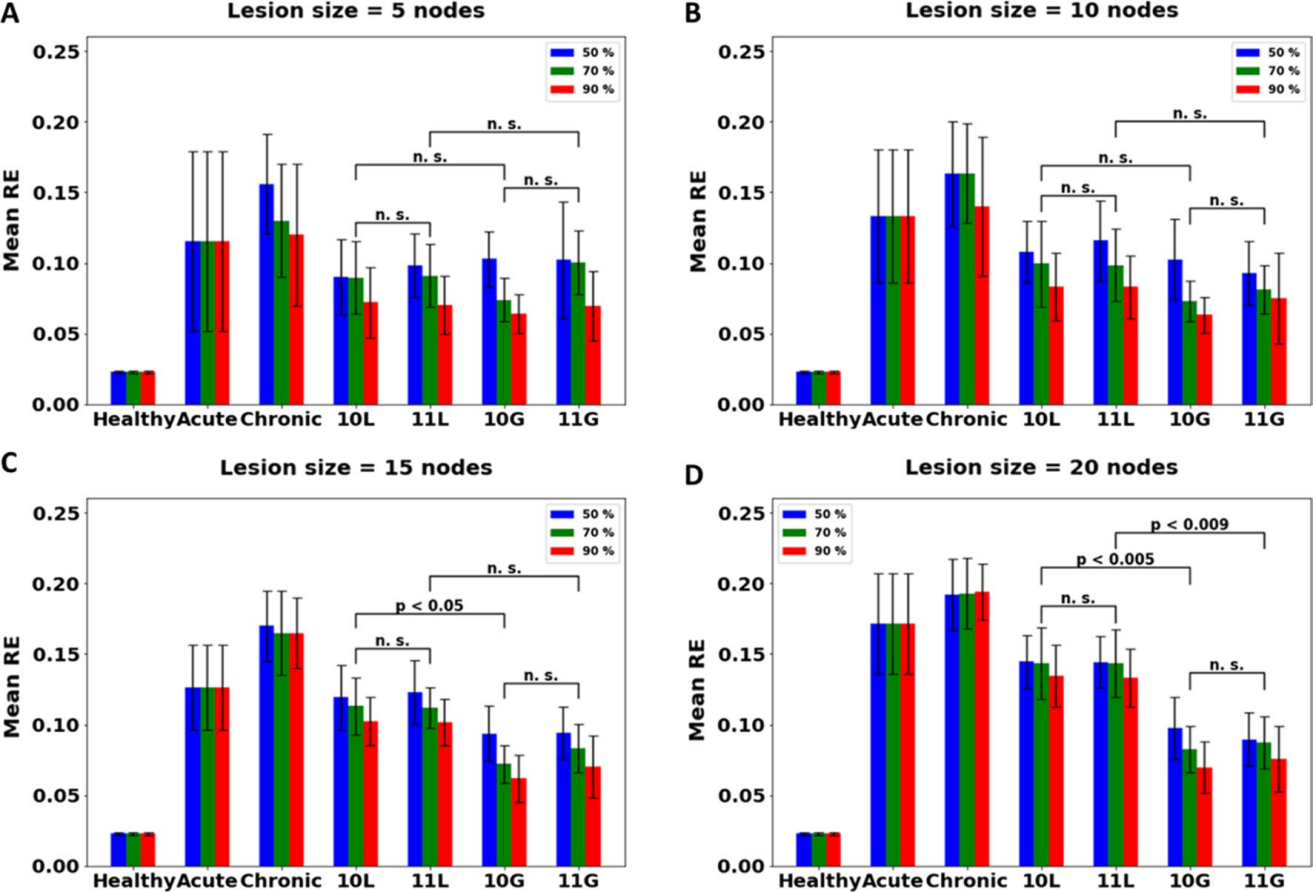
Comparison of different therapies for chronic stroke model with lesion size of **A** 5, **B** 10, **C** 15 and **D** 20, under different values of structural integrity of CC are given. Lesion is induced in the penultimate layer having a total of 30 nodes. [Key for x-axis: 10L (CIMT Exploratory under local plasticity), 11L (BMT Exploratory under local plasticity), 10G (CIMT Exploratory under global plasticity), 11G (BMT Exploratory under global plasticity)]. For lesion size of 5 and 10 nodes, the network does not show preference of one plasticity condition over the other (p > 0.1 under all CC integrity values when comparing 10L with 10G and 11L with 11G). However, for a lesion size of 15, 70% and 90% show preference of global plasticity over local plasticity (p < 0.05 when comparing 10L and 10 G, p > 0.1 when comparing 11L and 11G). However, for a lesion size of 20, regardless of the value of CC integrity, the network prefers global over local plasticity (p < 0.005 when comparing 10L and 10G for all CC integrity values, and p < 0.009 when comparing 10L and 10G). Thus, as the integrity of the connections goes down, the network tries to compensate with retraining of only the local connections, and preferring retraining of the entire network (global plasticity) only when the damage is too high (20 nodes)

Under acute condition, therapy is administered right after introducing lesion. However, for developing chronic stroke in the model, the model was made to perform unimanual movements with the healthy arm for a few epochs after inducing stroke. This condition can be taken as analogous to a patient who, after stroke onset, does not undergo rehabilitation of any kind until the end of acute stroke period. Thus, for chronic stroke, the model shows higher error when compared with acute stroke. The same therapies administered under acute condition are also used for the chronic models (Fig. 12). For 50% CC integrity under chronic models, similar to the acute model, the network seems to prefer local plasticity for lesion sizes 5, 10 and 15 (although for 10 and 15 the mean RE under global is lesser than the corresponding local, the difference is not significant, hence we recommend local plasticity for the network, as was the case with acute stroke condition), and global plasticity for a lesion size of 20 nodes (p < 0.005 for 10L vs 10G and p < 0.009 for 11L vs 11G). However, for 70% integrity, the network prefers local plasticity for a lesion size of 5 and 10 nodes (for lesion size of 5 and 10 the mean RE under global is lesser than the corresponding local, the difference is not significant, hence, again, we recommend local plasticity for the network), and global plasticity for 15 (p < 0.05 when comparing 10L and 10G) and 20 nodes (p < 0.005 for 10L vs 10G and p < 0.009 for 11L vs 11G). Similar result was also observed for 90% integrity (for a lesion size of n = 15, p < 0.05 when comparing 10L and 10G, and for lesion size of n = 20 p < 0.005 for 10L vs 10G and p < 0.009 for 11L vs 11G, while no significant difference was found (p > 0.1) between L and G therapies for lesion size of n = 5,10).

## Discussion

### Visuomotor network model

This paper describes a modified CNN capable of performing a visuomotor reaching task and used as a model for stroke-related motor impairment and rehabilitation. The CNN was designed as a network with bilateral symmetry to reflect the hemispherical organization of the brain. The resulting architecture was optimized to simulate a stereovision-based reaching task performed by humans. Whereas the convolutional layers closer to the input are interpreted as visual areas, the fully connected layers closer to the output are interpreted as motor areas. The cross-connections used in the network can be interpreted in terms of the corpus callosum fibres that connect the two hemispheres of the brain (Refer to Additional File 1 for further details related to this). We used this analogy to replicate the loss of structural integrity in the CC observed in stroke patients by setting the value of some of the cross-connection weights to zero. By doing this, we were able to illustrate the importance of structural properties of a network on recovery.

In order to introduce stroke in the optimized network, in line with what occurs in the brain after stroke onset, a few nodes in the motor region of the network were silenced. This affected the reaching performance of the network as quantified by mean RE. By observing the arm's workspace (Fig. 5), we see that the arm can reach to all the points in the workspace prior to stroke with seemingly no difficulty. However, post-stroke, the network loses its ability to reach to points accurately in specific regions. This region expands as the size of the lesion increases. After therapy, recovery is reflected by the network’s ability to regain the performance lost due to stroke. Thus, the network can serve as a simplified model of impairment and recovery post-stroke that is capable of incorporating properties of the lesion (lesion size, location, CC integrity) and the resulting impairment in functional properties of the arm of the affected individual (RE of the paretic arm).

### Measuring performance of the model

Reaching Error (RE) is the parameter used in the study to analyse the performance of the network under normal and stroke conditions. Both healthy, and impaired models are analysed using this parameter. In the clinical setting, motor impairment after stroke is measured with the help of standardised tests like Fugl Meyer Analysis (FMA), Wolf Motor Function Test (WMFT) etc. However, there are several disadvantages associated with these measures. For instance, they do not always make a distinction between recovery and compensation, and are very subjective in nature since they are scored by visual observation performed by physiotherapists. Hence, several attempts have been made by researchers over the years to invent better measures to evaluate recovery after stroke. Some of these include kinematic parameters such as movement time, smoothness of trajectory, peak velocity, and accuracy [34, 35]. Accuracy can be measured by both trajectory length, and endpoint accuracy. RE has been used by multiple studies to measure recovery and the improvements in RE have also been correlated with improvement in standard scales used in stroke [36–39]. RE measures the distance between the final hand point and the target position, which are distributed all over the workspace, thereby determining the accuracy of the intended movement. Additionally, in a recent consensus paper authored by multiple stroke experts regarding the kinematic measures to be used in place of standardised scales for measuring impairment, endpoint accuracy was a suggested parameter [40]. Since RE can be readily measured by our model, we chose this as our measure of performance.

### Promoting recovery under acute stroke

In the current study, we capture two stages in stroke recovery—acute and chronic stages. In patients, the acute stage occurs after the onset of stroke and lasts up to 2 weeks, after which the subacute period starts and lasts up to six months. Chronic stage sets in after this and can last for the patient’s entire lifetime [41]. In order to replicate the acute stage and the subsequent therapy session in the model, we initiate the retraining of the model directly after introducing the lesion. The model undergoes therapy under two different movement complexity conditions—Exploratory vs. Stereotypic—and two different movement types differing in hand selection mode—CIMT vs. BMT. Conventional therapy administered in the form of occupational therapy is often set up to replicate tasks performed during Activities of Daily Life (ADL) to restore the patient’s independence in their daily life [42]. Thus, the movements used are related to the tasks performed by the arms during a typical day [43]. Stereotypic movement condition captures this in the restricted area of its workspace while exploratory movement condition is set up in contrast by using the entire workspace. Upon administering the four combinations, we find an apparent bias of the model to recover better under the Exploratory condition when compared with the Stereotypic condition, regardless of the type of movement used (CIMT or BMT). Due to the contradictory approaches between CIMT and BMT, there has long been a debate about deciding the more efficient therapy in promoting recovery for hemiparesis, with no clear winner emerging [44]. The results obtained in this study seem to show that the movement complexity (Stereotypic vs Exploratory) chosen for therapy is more important than the hand selection mode as long as the affected arm is moved (Fig. 8 for acute and Fig. 9 for chronic). Strengthening this point further, we see this result repeated across the results discussed here, with the Exploratory approach being significantly better than Stereotypic for both acute and chronic models under local and global plasticity conditions regardless of level of CC integrity. In the clinical setting, patients often find it difficult to move their arm more than a few inches. For such patients, Exploratory therapy might not be a realistic rehabilitation technique. During the course of the rehabilitation, patients start with simpler movements and then progress to more difficult ones which slowly take them away from their comfort zone [45, 46]. This can be modified such that, the patients expand their comfort zone in both the action space and the workspace, slowly incorporating more “exploratory” type movements. Supplementing such rehabilitation practices along with the conventional standard therapy in use could improve performance to levels more than what is typically observed in patients.

### Overcoming learned non-use in chronic stroke

During the chronic stage, many patients suffering from hemiparesis caused due to stroke undergo a phenomenon of learned non-use, whereby due to the prolonged impairment and reduced functionality of the paretic arm, the patients tend to use healthy arm as a compensation in ADL. This in turn leads to the setting up of a vicious cycle of non-use due to impairment, and worsening impairment due to non-use leading to further non-use [28]. While there are many reasons for why the patients choose the healthy arm over the paretic arm to make movements, these are not considered in this study—only the effects of the phenomenon are considered, and not the causes [44]. CIMT is meant to counteract the effect caused by learned non-use, by restraining the unaffected healthy arm while encouraging the use of the affected paretic arm [9]. In order to replicate the effect of non-use and induce chronic stroke in the model, instead of retraining with therapy directly after inducing stroke, we run the model over a few epochs of retraining where we choose movements such that only the healthy arm is moved. Thus, this induces learned non-use in the model, and it is reflected in the deteriorating performance of the paretic arm. Furthermore, from the model, we can also see that for larger lesion sizes, setting up learned non-use proves to be dangerous as performance gets worse than acute stroke. Once the chronic model is created, therapy is administered in a manner similar to the acute stroke models. Here, again, we see that in the Exploratory condition performance is better than under the Stereotypic condition regardless of the hand selection mode (CIMT or BMT) used for therapy. However, upon comparison with the acute models, the recovery observed is not comparable to the healthy condition, even for smaller lesion sizes. This is similar to what is observed in reality, as chronic patients do not show same improvement as acute patients.

In order to improve recovery in the model, we chose to increase the number of retrained connections by retraining the weights in the entire model (global plasticity) instead of only retraining the weights associated with the lesioned layer (local plasticity) i.e., increasing the extent of plasticity. This allowed us to analyse the effect the number of retrained connections had on recovery. Implementing the previously used combinations of therapy, we see that under global plasticity condition, the performance of the network is significantly better than using local plasticity condition for all lesion sizes. Again, similar to what we observed in the acute models, we can see that Exploratory is better than using Stereotypic movements. Interestingly, in the acute models, with 100% CC integrity, we observed that the model showed lesion size-specific improvement, i.e., for smaller lesion sizes, local and global plasticity showed similar improvement while for larger lesion sizes global plasticity was better at promoting recovery. This lesion size-specific preference was not observed in the chronic models. Regardless of the size of the lesion, the chronic models showed better performance under global plasticity condition for 100% CC integrity. Thus, the network showed preference to an increased extent of plasticity as the damage to the network worsened.

### Multisensory integration as rehabilitation therapy

According to the proportional recovery rule (PRR), most patients achieve 70% recovery in the first three months after stroke [47]. This time period is often called as the “critical period” and is characterized by heightened plasticity opened by the injury caused due to stroke [48]. After this period, especially once the patient enters the chronic phase, recovery is often minimal. Furthermore, patients in the acute and subacute stage show recovery proportional to the dosage of therapy (number of sessions, number of repetitions of movement per session, etc.) used [49]. However, this phenomenon is not exhibited in chronic stroke patients [50]. In order to help in the recovery of chronic patients, several researchers have tried to restart, and boost plasticity in the perilesional areas as a way of reopening the critical period [51–53]. Recently, studies in rodents [33] have shown that using Enriched Environment (EE), which can promote multimodal sensory stimulation, can be beneficial for recovery. This is possible because multi-sensory stimulation can activate areas functionally connected to the regions affected by stroke and increase plasticity at the perilesional site. Similar studies have also been proposed in humans by using gaming environments, simulated in Virtual Reality (VR). These environments can serve as EE by providing an immersive experience as therapy [15]. Using an EE helps in both engaging a patient during therapy, by keeping their curiosity and motivational levels high and at the same time, activating more areas in the brain. This specific feature of the EE is what we try to achieve with the help of global plasticity in this study, wherein we also try to recruit more areas, and make the network more conducive to the therapy offered. On the other hand, local plasticity refers to using conventional therapy for rehabilitation which focuses only on repeated performance of motor tasks. The results obtained here indicate that such therapies similar to EE need to be explored more, especially for chronic stroke patients.

### Role of corpus callosum in recovery after stroke

**Fig. 13:**
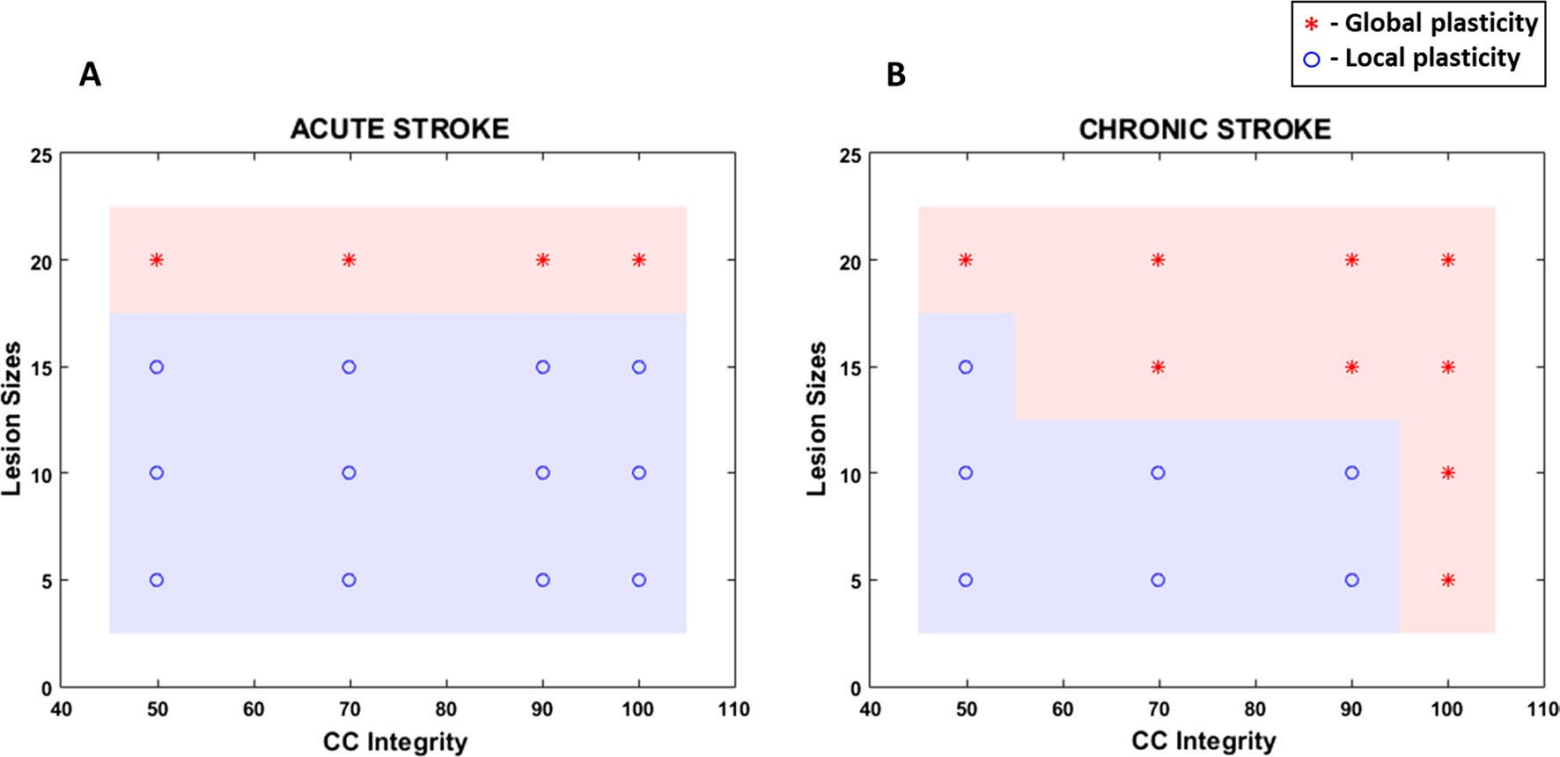
Plot showing regions of optimal plasticity condition for **A** acute stroke and **B** chronic stroke

In the study discussed here, we were able to demonstrate how a simple convolutional network trained on supervised learning can function as a simplified model of recovery post-stroke. The network properties were manipulated in order to incorporate several features of stroke like time since onset (acute vs chronic), location of the lesion, size of the lesion and also damage caused to the integrity of the neighbouring regions like the CC due to the lesion. By including these as characteristics in the study, we were able to understand the impact of each of these features on post-stroke recovery. When no significant difference was found between the two plasticity conditions, considering the computational cost involved, local plasticity was recommended for the network. Translating this result to a clinical setting, since no significant difference is found between the two plasticity conditions, and keeping in mind that (1) approximately same level of recovery is achieved in both, (2) global plasticity-type rehabilitation requires additional factors such as EE, VR, multisensory integration etc., local plasticity-type rehabilitation (i.e., conventional therapy) maybe recommended. Based on this, the plasticity condition recommended for different levels of CC integrity and different lesions sizes, under acute and chronic stroke, are illustrated in Fig. 13.

In the model, for acute stroke condition, CC integrity does not play a role in the preference of the plasticity condition. For all CC integrity values, if the lesion size is 20 (highest lesion size considered), the model prefers global over local plasticity. However, for chronic stroke condition, the network shows the effect of CC integrity on the preference of the plasticity condition. Comparing the damage caused by a given lesion size, in a model with a given level of CC integrity, the model with chronic stroke experiences higher performance loss than the same model with acute stroke. Thus, to regain the lost performance, a chronic model would likewise require more effort. In the current study, regardless of the level of damage, all models are given rehabilitation with the same number of points over the same number of training epochs. This was done in order to bring out the differences in setting (i.e., plasticity condition required, and type of workspace required) required for performance to improve in both the models. We believe that the extra effort required by the chronic model is reflected in the plasticity preference since global plasticity recruits more connections and resources during training when compared to local plasticity. Hence chronic models prefer global plasticity more than acute models. Bringing this result to the experimental setting, in a stroke study with mice [54], the authors were able to show that by inducing a second stroke to the mice, the window of plasticity occurring after stroke can be reopened and helps in the recovery of damage caused by both the strokes. Thus, plasticity plays a very important role in recovery after stroke. Obviously, it is not suggested that a stroke patient be induced with a second stroke. However, other ways to induce plasticity needs to be explored to promote efficient recovery.

The role of the cortico-spinal tract in impact due to, and recovery after, stroke has long been measured in clinical studies. However, only recently the effect of CC has been studied [30, 31]. It is often found that the level of CC integrity even in controls can vary due to many different lifestyle choices, fitness levels [55], pre-morbidities like diabetes [56, 57] and sometimes even hobbies like playing a musical instrument [58, 59]. The study discussed here illustrates the necessity to understand and profile a patient completely before prescribing a rehabilitation protocol for them. In such scenarios, a computational model can come in handy by serving as a test bed that can combine different features of the patient and reveal the optimal rehabilitation protocol suitable for that patient. The model discussed here, though simple from a computational perspective and obviously not without limitations (discussed below), can serve as a prototype for such a test bed and also offers testable predictions and hypotheses that can be relevant to stroke rehabilitation.

## Conclusion

The main conclusions from the study reported here are given below:

*Effect of Movement Complexity:* Upon comparing recovery from using a Stereotypic vs using Exploratory movements, it is clear that Exploratory movements are much better in facilitating recovery. This is seen across models regardless of the size of the lesion, number of connections available for retraining, time since stroke onset (acute/chronic) and structural integrity. Thus, this result illustrates that more emphasis should be given on designing the environment used for rehabilitation such that it is equipped to encourage patients to move and explore.

*Effect of Hand Selection Mode:* To the stroke model, two types of movements were administered in the form of therapy—BMT and CIMT. However, upon comparison of recovery between these two, there emerged no significant difference.

*Effect of Plasticity:* It is seen from the study here that under more debilitating conditions like higher lesion size (for acute model) or longer time since stroke onset (chronic condition), increasing number of connections available for retraining results in significant improvements. In the clinical setting, increasing the number of connections can translate to activating the intact regions of the brain by using techniques like multisensory integration.

*Effect of Structural Integrity:* The structural integrity of the network plays an important role in recovery. Even with high levels of damage (higher lesion size under acute condition, or if the network is in chronic stroke condition) if the structural integrity is compromised, the network prefers local plasticity over the global plasticity condition in restoring performance.

Although CNNs have been used before to model the sensory systems of the brain, this is the first time, to our knowledge, they have been applied to simulate the visuomotor pathway of the brain, particularly to model stroke rehabilitation. The model is capable of replicating a simple bimanual visuomotor reaching task in 3D space and the impairment caused by a stroke in the motor cortex along with the subsequent recovery after administration of therapy. However, at present, it does have several limitations.

- The role of the ipsilateral hemisphere in controlling the upper extremity function is not considered in the model, even though substantial evidence exists for the control of the arm function by M1 of the ipsilateral hemisphere [60, 61].
- The current model only shows a gross similarity with the visuomotor pathway of the brain. However, the modelling efforts discussed in this study are part of a larger project involving clinical, and imaging aspects. Hence, in future versions of the model, efforts will be made to make the model to more closely resemble the brain, in order to draw comparisons between imaging data obtained from patients, and the activity pattern obtained in the network.
- Only lesion in the penultimate layer is discussed, no other lesion location is considered. This also needs to be done after the brain-network mapping is performed.
- Arm setup used cannot show dynamic features like force, rigidity etc. Expanding the muscle model to incorporate these can help in also understanding the effect of stroke on these parameters which are well established in clinical studies.
- Additionally, since the current model is a static model, movement complexity, which is used as one of the characteristics used to define therapy, can only be defined in terms of the range of the workspace covered. But if a dynamic network is used, movement complexity can also be defined in terms of the complexity of the trajectory. In a future work, we intend to do the same.
- Furthermore, providing feedback on the movement made by visually showing the patient's hand movement has proven to be quite effective for stroke rehabilitation [62]. Currently, since the model only performs one-shot reaching, therapy with feedback cannot be administered.
- A more rigorous comparison between the network model and real clinical setting in the number of epochs used for therapy, time since onset and difference in arm use under acute and chronic condition (by including the nature and the amount of therapy administered) can help in providing rehabilitation protocol that can be directly used on the patient.

Thus, the aforementioned limitations should be addressed in a future study. Expanding the network to include such dynamic aspects can help in developing the model into a more realistic and patient-specific test bench to provide the optimal rehabilitation protocol in a patient-specific fashion.

## Supplementary Information

**Additional file 1:** Network Architecture Optimization.

**Additional file 2.** Statistical Analysis.

## Data Availability

The datasets used and/or analysed during the current study are available from the corresponding author on reasonable request.

## Abbreviations

CIMT: Constraint-induced movement therapy
BMT: Bimanual movement therapy
CNN: Convolutional neural network
MN_AG_: Muscle activation for agonist
MN_AN_: Muscle activation for antagonist
H_0_: Starting position of hand
E_0_: Starting position of elbow
S_0_: Starting position of shoulder
${\mathrm{H}}_{\mathrm{e}}^{\mathrm{R}/\mathrm{L}}$: Final position of hand (right or left)
HSP: Hand selection parameter
RE: Reaching error
CC: Corpus callosum
ADL: Activities of daily living
PRR: Proportional recovery rule
EE: Enriched environment
VR: Virtual reality

## References

[1] Mozaffarian D, Benjamin EJ, Go AS, Arnett DK, Blaha MJ, Cushman M (2016). Heart disease and stroke statistics-2016 update a report from the American Heart Association. Circulation.

[2] Donkor ES (2018). Stroke in the 21st century: a snapshot of the burden, epidemiology, and quality of life. Stroke Res Treat.

[3] Faria-Fortini I, Michaelsen SM, Cassiano JG, Teixeira-Salmela LF (2011). Upper extremity function in stroke subjects: relationships between the international classification of functioning, disability, and health domains. J Hand Ther.

[4] Cramer SC, Nelles G, Benson RR, Kaplan JD, Parker RA, Kwong KK (1997). A functional MRI study of subjects recovered from hemiparetic stroke. Stroke.

[5] Samsa GP, Matchar DB (2004). How strong is the relationship between functional status and quality of life among persons with stroke?. J Rehabil Res Dev.

[6] Miller EL, Murray L, Richards L, Zorowitz RD, Bakas T, Clark P (2010). Comprehensive overview of nursing and interdisciplinary rehabilitation care of the stroke patient: a scientific statement from the American Heart Association. Stroke.

[7] Schaechter JD (2004). Motor rehabilitation and brain plasticity after hemiparetic stroke. Prog Neurobiol.

[8] Pollock A, Baer G, Campbell P, Choo PL, Forster A, Morris J (2014). Physical rehabilitation approaches for the recovery of function and mobility following stroke. Cochrane Database Syst Rev.

[9] Wolf SL, Lecraw DE, Barton LA, Jann BB (1989). Forced use of hemiplegic upper extremities to reverse the effect of learned nonuse among chronic stroke and head-injured patients. Exp Neurol.

[10] Rose DK, Winstein CJ (2004). Bimanual training after stroke: are two hands better than one?. Top Stroke Rehabil.

[11] Rensink M, Schuurmans M, Lindeman E, Hafsteinsdóttir T (2009). Task-oriented training in rehabilitation after stroke. J Adv Nurs.

[12] Sorinola IO, Fergusson M, Skevington-Postles L (2016). The effect of rehabilitation interventions on long term upper limb function in chronic stroke patients: a meta-analysis. Physiotherapy.

[13] Krakauer JW (2006). Motor learning: its relevance to stroke recovery and neurorehabilitation. Curr Opin Neurol.

[14] Shea JB, Morgan RL (1979). Contextual interference effects on the acquisition, retention, and transfer of a motor skill. J Exp Psychol Hum Learn.

[15] Krakauer JW, Cortés JC (2018). A non-task-oriented approach based on high-dose playful movement exploration for rehabilitation of the upper limb early after stroke: a proposal. NeuroRehabilitation.

[16] Krakauer JW, Kitago T, Goldsmith J, Ahmad O, Roy P, Stein J (2021). Comparing a novel neuroanimation experience to conventional therapy for high-dose intensive upper-limb training in subacute stroke: the SMARTS2 Randomized Trial. Neurorehabil Neural Repair.

[17] Reinkensmeyer DJ, Burdet E, Casadio M, Krakauer JW, Kwakkel G, Lang CE (2016). Computational neurorehabilitation: modeling plasticity and learning to predict recovery. J Neuroeng Rehabil.

[18] Chen Y, Reggia JA. Alignment of Coexisting Cortical Maps in a Motor Control Model. n.d. 10.1162/neco.1996.8.4.731 8624960

[19] Han CE, Arbib MA, Schweighofer N (2008). Stroke rehabilitation reaches a threshold. PLoS Comput Biol.

[20] Narayanamurthy R, Jayakumar S, Elango S, Muralidharan V, Chakravarthy VS (2019). A cortico- basal ganglia model for choosing an optimal rehabilitation strategy in hemiparetic stroke. Sci Rep.

[21] Takiyama K, Okada M. Recovery in Stroke Rehabilitation through the Rotation of Preferred Directions Induced by Bimanual Movements: A Computational Study n.d. 10.1371/journal.pone.0037594. 10.1371/journal.pone.0037594 PMC3360015 22655060

[22] Kwakkel G, Kollen BJ (2013). Predicting activities after stroke: what is clinically relevant?. Int J Stroke.

[23] Stinear C (2010). Prediction of recovery of motor function after stroke. Lancet Neurol.

[24] Kell AJ, McDermott JH (2019). Deep neural network models of sensory systems: windows onto the role of task constraints. Curr Opin Neurobiol.

[25] Kell AJE, Yamins DLK, Shook EN, Norman-Haignere SV, McDermott JH (2018). A task-optimized neural network replicates human auditory behavior, predicts brain responses, and reveals a cortical processing hierarchy. Neuron.

[26] Features — blender.org n.d. https://www.blender.org/features/ (accessed March 29, 2022).

[27] 2.80 — blender.org n.d. https://www.blender.org/download/releases/2-80/ (accessed March 29, 2022).

[28] The learned nonuse phenomenon: implications for rehabilitation - Europa Medicophysica 2006 September;42(3):241–55 - Minerva Medica - Journals n.d. https://www.minervamedica.it/en/journals/europa-medicophysica/article.php?cod=R33Y2006N03A0241 (accessed March 30, 2022). 17039223

[29] Ballester BR, Maier M, Segundo RS, Galeano VC, Duff A, Verschure PFMJ. Reinforcement-induced movement therapy: A novel approach for overcoming learned non-use in chronic stroke patients. International Conference on Virtual Rehabilitation, ICVR 2015:183–90. 10.1109/ICVR.2015.7358586.

[30] Stewart JC, O’Donnell M, Handlery K, Winstein CJ (2017). Skilled reach performance correlates with corpus callosum structural integrity in individuals with mild motor impairment after stroke: a preliminary investigation. Neurorehabil Neural Repair.

[31] Stewart JC, Dewanjee P, Tran G, Quinlan EB, Dodakian L, McKenzie A (2017). Role of corpus callosum integrity in arm function differs based on motor severity after stroke. Neuroimage Clin.

[32] Sisti HM, Geurts M, Gooijers J, Heitger MH, Caeyenberghs K, Beets IAM (2012). Microstructural organization of corpus callosum projections to prefrontal cortex predicts bimanual motor learning. Learn Mem.

[33] Hakon J, Quattromani MJ, Sjölund C, Tomasevic G, Carey L, Lee JM (2018). Multisensory stimulation improves functional recovery and resting-state functional connectivity in the mouse brain after stroke. Neuroimage Clin.

[34] do Tran V, Dario P, Mazzoleni S (2018). Kinematic measures for upper limb robot-assisted therapy following stroke and correlations with clinical outcome measures: a review. Med Eng Phys.

[35] Villepinte C, Verma A, Dimeglio C, de Boissezon X, Gasq D (2021). Responsiveness of kinematic and clinical measures of upper-limb motor function after stroke: a systematic review and meta-analysis. Ann Phys Rehabil Med.

[36] Gilliaux M, Lejeune TM, Detrembleur C, Sapin J, Dehez B, Selves C (2014). Using the robotic device REAplan as a valid, reliable, and sensitive too l to quantify upper limb impairments in stroke patients. J Rehabil Med.

[37] Goffredo M, Mazzoleni S, Gison A, Infarinato F, Pournajaf S, Galafate D (2019). Kinematic parameters for tracking patient progress during upper limb robot-assisted rehabilitation: an observational study on subacute stroke subjects. Appl Bionics Biomech.

[38] Lang CE, Wagner JM, Dromerick AW, Edwards DF (2006). Measurement of upper-extremity function early after stroke: properties of the action research arm test. Arch Phys Med Rehabil.

[39] Duret C, Courtial O, Grosmaire AG (2016). Kinematic measures for upper limb motor assessment during robot-mediated training in patients with severe sub-acute stroke. Restor Neurol Neurosci.

[40] Kwakkel G, van Wegen EEH, Burridge JH, Winstein CJ, van Dokkum LEH, Alt Murphy M (2019). Standardized measurement of quality of upper limb movement after stroke: consensus-based core recommendations from the second stroke recovery and rehabilitation roundtable. Neurorehabil Neural Repair.

[41] Kiran S (2012). What is the nature of poststroke language recovery and reorganization?. ISRN Neurol.

[42] Legg LA, Lewis SR, Schofield-Robinson OJ, Drummond A, Langhorne P (2017). Occupational therapy for adults with problems in activities of daily living after stroke. Cochrane Database Syst Rev.

[43] Howard IS, Ingram JN, Körding KP, Wolpert DM (2009). Statistics of natural movements are reflected in motor errors. J Neurophysiol.

[44] Stoykov ME, Lewis GN, Corcos DM (2009). Comparison of bilateral and unilateral training for upper extremity hemiparesis in stroke. Neurorehabil Neural Repair.

[45] Woldag H, Stupka K, Hummelsheim H (2010). Repetitive training of complex hand and arm movements with shaping is beneficial for motor improvement in patients after stroke. J Rehabil Med.

[46] Ballester BR, Maier M, San Segundo Mozo RM, Castañeda V, Duff A, Verschure PF (2016). Counteracting learned non-use in chronic stroke patients with reinforcement-induced movement therapy. J Neuroeng Rehabil.

[47] Krakauer JW, Carmichael ST, Corbett D, Wittenberg GF (2012). Getting neurorehabilitation right: what can be learned from animal models?. Neurorehabil Neural Repair.

[48] Dromerick AW, Geed S, Barth J, Brady K, Giannetti ML, Mitchell A (2021). Critical Period After Stroke Study (CPASS): A phase II clinical trial testing an optimal time for motor recovery after stroke in humans. Proc Natl Acad Sci USA.

[49] Lohse KR, Lang CE, Boyd LA (2014). Is more better? Using metadata to explore dose-response relationships in stroke rehabilitation. Stroke.

[50] Lang CE, Strube MJ, Bland MD, Waddell KJ, Cherry-Allen KM, Nudo RJ (2016). Dose response of task-specific upper limb training in people at least 6 months poststroke: a phase II, single-blind, randomized, controlled trial. Ann Neurol.

[51] Ng KL, Gibson EM, Hubbard R, Yang J, Caffo B, O’Brien RJ (2015). Fluoxetine maintains a state of heightened responsiveness to motor training early after stroke in a mouse model. Stroke.

[52] Chollet F, Tardy J, Albucher JF, Thalamas C, Berard E, Lamy C (2011). Fluoxetine for motor recovery after acute ischaemic stroke (FLAME): a randomised placebo-controlled trial. Lancet Neurol.

[53] Jones TA, Adkins DL (2015). Motor system reorganization after stroke: stimulating and training toward perfection. Physiology (Bethesda).

[54] Zeiler SR, Hubbard R, Gibson EM, Zheng T, Ng K, O’Brien R (2016). Paradoxical motor recovery from a first stroke after induction of a second stroke: re-opening a post-ischemic sensitive period. Neurorehabil Neural Repair.

[55] Loprinzi PD, Harper J, Ikuta T (2020). The effects of aerobic exercise on corpus callosum integrity: systematic review. Phys Sportsmed.

[56] Antenor-Dorsey JA, Meyer E, Rutlin J, Perantie DC, White NH, Arbelaez AM (2013). White matter microstructural integrity in youth with type 1 diabetes. Diabetes.

[57] Reijmer YD, Brundel M, de Bresser J, Kappelle LJ, Leemans A, Biessels GJ (2013). Microstructural white matter abnormalities and cognitive functioning in type 2 diabetes: a diffusion tensor imaging study. Diabetes Care.

[58] Schlaug G, Forgeard M, Zhu L, Norton A, Norton A, Winner E (2009). Training-induced neuroplasticity in young children. Ann N Y Acad Sci.

[59] Lee DJ, Chen Y, Schlaug G (2003). Corpus callosum: musician and gender effects. NeuroReport.

[60] Ganguly K, Secundo L, Ranade G, Orsborn A, Chang EF, Dimitrov DF (2009). Cortical representation of ipsilateral arm movements in monkey and man. J Neurosci.

[61] Brus-Ramer M, Carmel JB, Martin JH (2009). Motor cortex bilateral motor representation depends on subcortical and interhemispheric interactions. J Neurosci.

[62] Grimm F, Naros G, Gharabaghi A (2016). Compensation or restoration: Closed-loop feedback of movement quality for assisted reach-to-grasp exercises with a multi-joint arm exoskeleton. Front Neurosci.

