# Supplementary material for: Interaction of network and rehabilitation therapy parameters in defining recovery after stroke in a Bilateral Neural Network
Source: J Neuroeng Rehabil. 2022 Dec 19;19:142. doi: 10.1186/s12984-022-01106-3 (PMC9762011; doi:10.1186/s12984-022-01106-3)
Supplement: Supplementary file 1 — Additional file 1: Network Architecture Optimization. [file 12984_2022_1106_MOESM1_ESM.docx]

Additional File 1

Network Architecture Optimization

# Method

# A simple convolutional neural network (CNN) model was chosen to replicate the visuomotor pathway for our study (schematic is shown in fig. 1). The network was split vertically into two parts in order to account for the bilateral nature of the human body. Each half received retinal image input as seen by either left or right eye and the output from the same half controlled the corresponding arm.


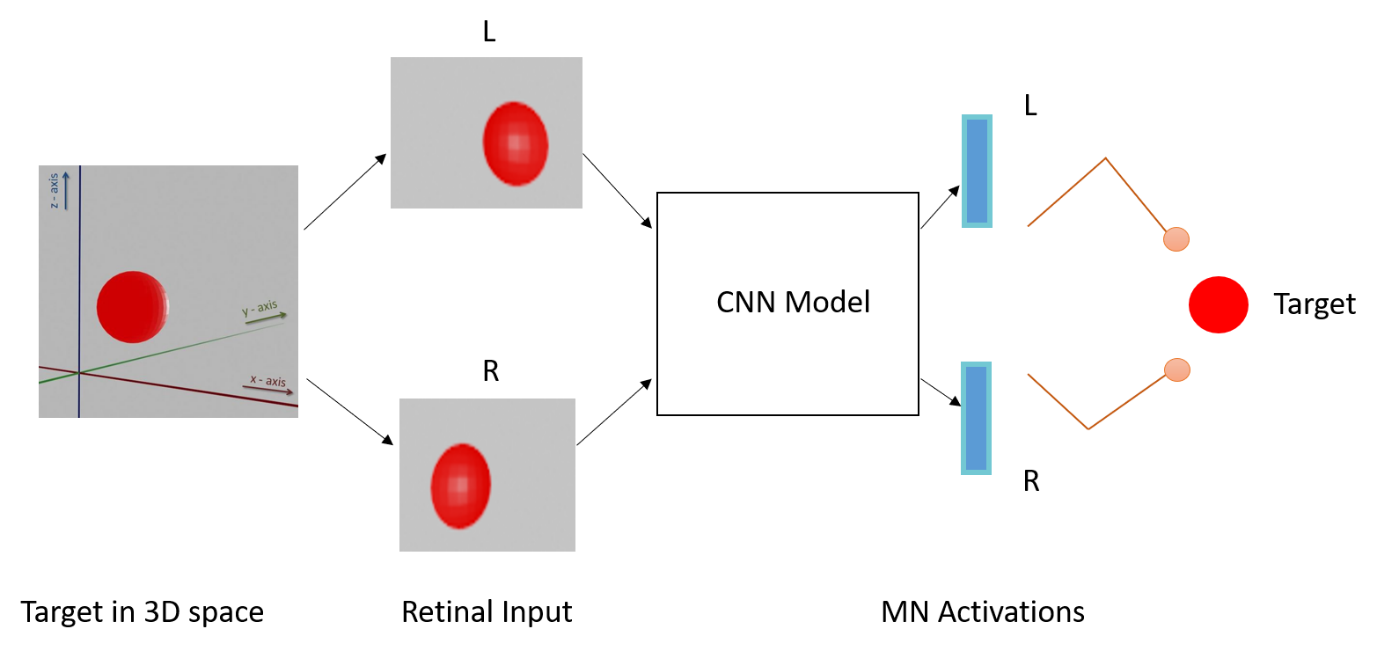


**Fig 1.** Schematic diagram of the proposed model.

# The network consisted of 5 convolutional layers and 3 fully connected layers. We started with a simple convolutional network consisting of 3 convolutional layers and 2 fully connected layers and modified the number of layers according to the training error. Details about the different parameters of the network can be found in Table 1.

| **Layer no. and type** | **Activation function used** | **Regularization used** | **No. of feature maps/nodes** | **Kernel Size** | **Stride length** | **Pooling size** | **Stride length** |
| --- | --- | --- | --- | --- | --- | --- | --- |
| 1 (Convolutional Layer) | Relu | L2 | 2+2 | (5,5) | (1,1) | (2,2) | (2,2) |
| 2 (Convolutional Layer) | Relu | L2 | 4+4 | (5,5) | (1,1) | - | - |
| 3 (Convolutional Layer) | Relu | L2 | 8+8 | (5,5) | (1,1) | - | - |
| 4 (Convolutional Layer) | Relu | L2 | 4+4 | (5,5) | (1,1) | - | - |
| 5 (Convolutional Layer) | Relu | L2 | 2+2 | (5,5) | (1,1) | - | - |
| 6 (Fully connected layer) | Sigmoid | L2 | 50+50 | - | - | - | - |
| 7 (Fully connected layer) | Sigmoid | L2 | 30+30 | - | - | - | - |
| 8(Fully connected layer) | Sigmoid | L2 | 6+6 | - | - | - | - |

**Table 1.** Hyperparameters used in the network.

# The connections between two successive layers within a single half of the network (ipsilateral connections) was provided at all layers. Whereas, connections between the two halves (contralateral connections – cross-connections) was optimized such that it was only provided in a few layers. The following studies were conducted in order to optimize the network.

# 1. Removing cross-connections in the entire network –

# The cross-connections in the network as a whole were removed (Absent or set to 0) while maintaining complete connectivity at the ipsilateral side (fig. 2. b). The performance of the network, under this condition was then contrasted with having full set of cross-connections (Present) on the network (fig. 2. a).


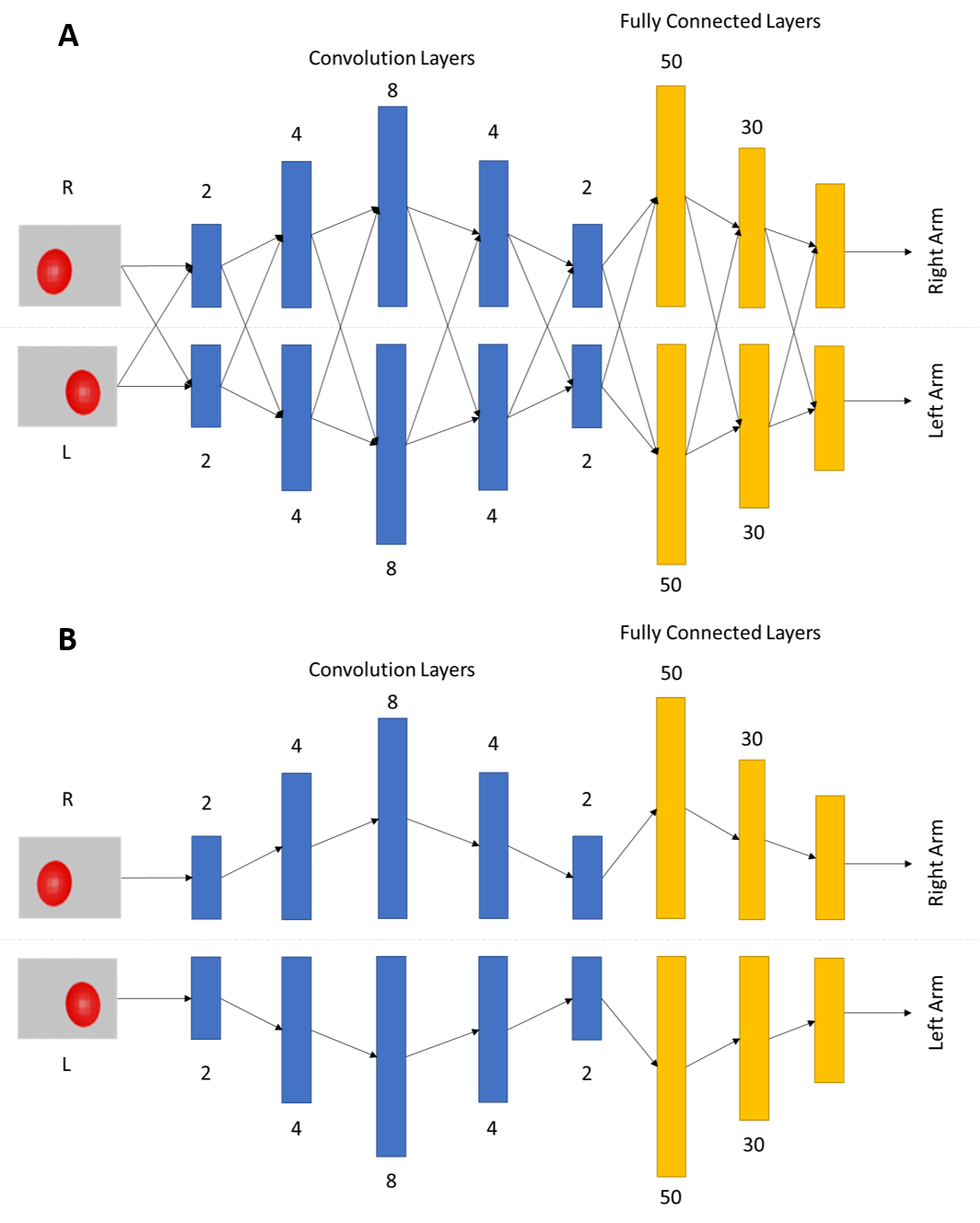


**Fig 2.** Network configuration upon removing cross-connections from the entire network A. All the layers contain cross-connections (Present condition), B. None of the layers contain cross- connections (Absent condition).

# 2. Removing cross-connections in different parts of the network –

# In addition to splitting the network based on laterality, the network was classified based on their functionality as Visual and Motor Regions. The network's convolutional layers were collectively termed the Visual Region (shown as blue bars in fig. 3), and the fully connected layers were termed the Motor Region (shown as yellow bars in fig. 3). The cross-connections in one region (visual or motor) of the network were fixed (set at either Absent or Present) and the connections on the other region were varied (set at either Absent or Present). This was done to check if the network preferred cross-connection in one of the regions – visual or motor – over the other. Performance of the network was assessed for each of the four combinations.


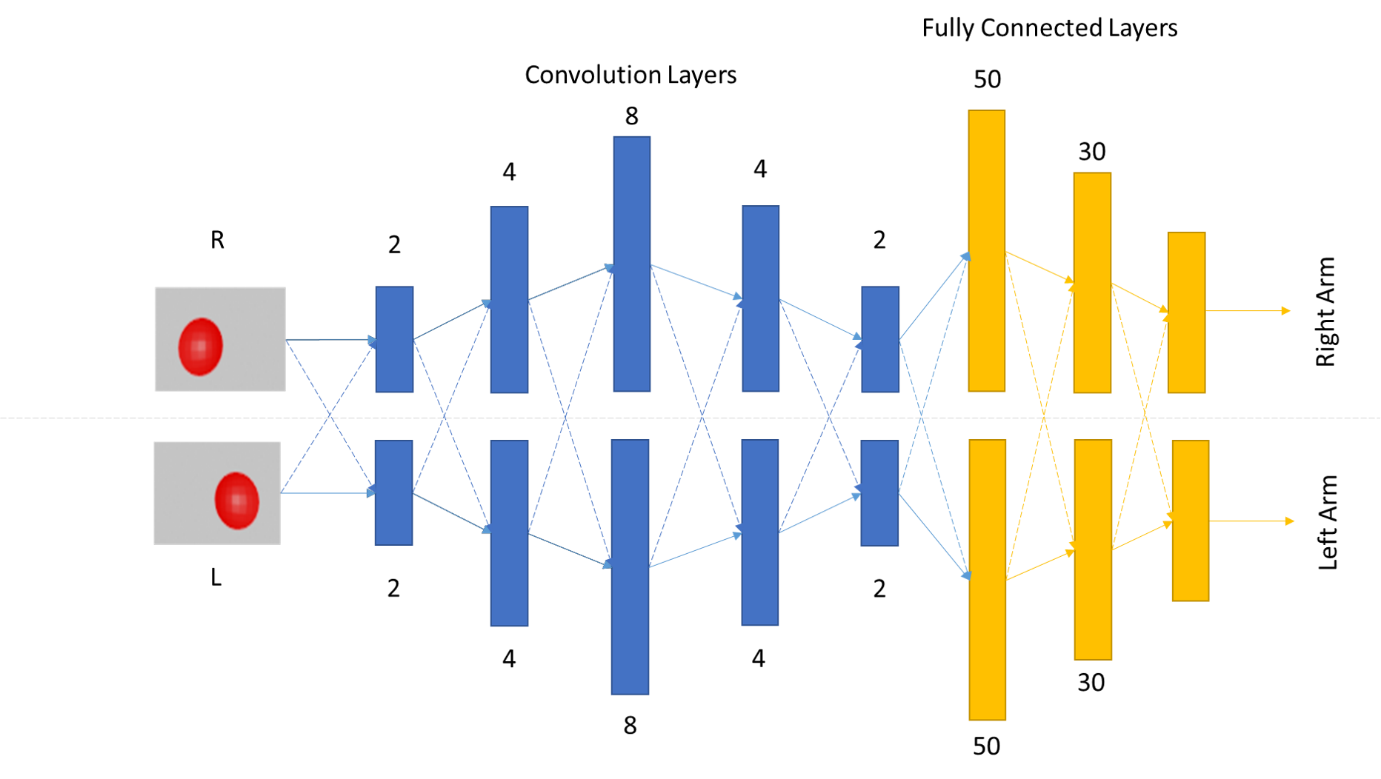


**Fig 3.** Network layers divided into two regions – Visual Region (Blue) and Motor Region (Yellow)

# 3. Removing cross-connections in stages –

# Under this condition, the cross-connections between the network layers were set to zero, starting either from the visual (input) (schematic shown in fig. 5) or motor (output) (schematic shown in fig. 4) regions until all the cross-connections were removed. The difference in effect while removing the connection from one side to the other was quantified with the help of RE.


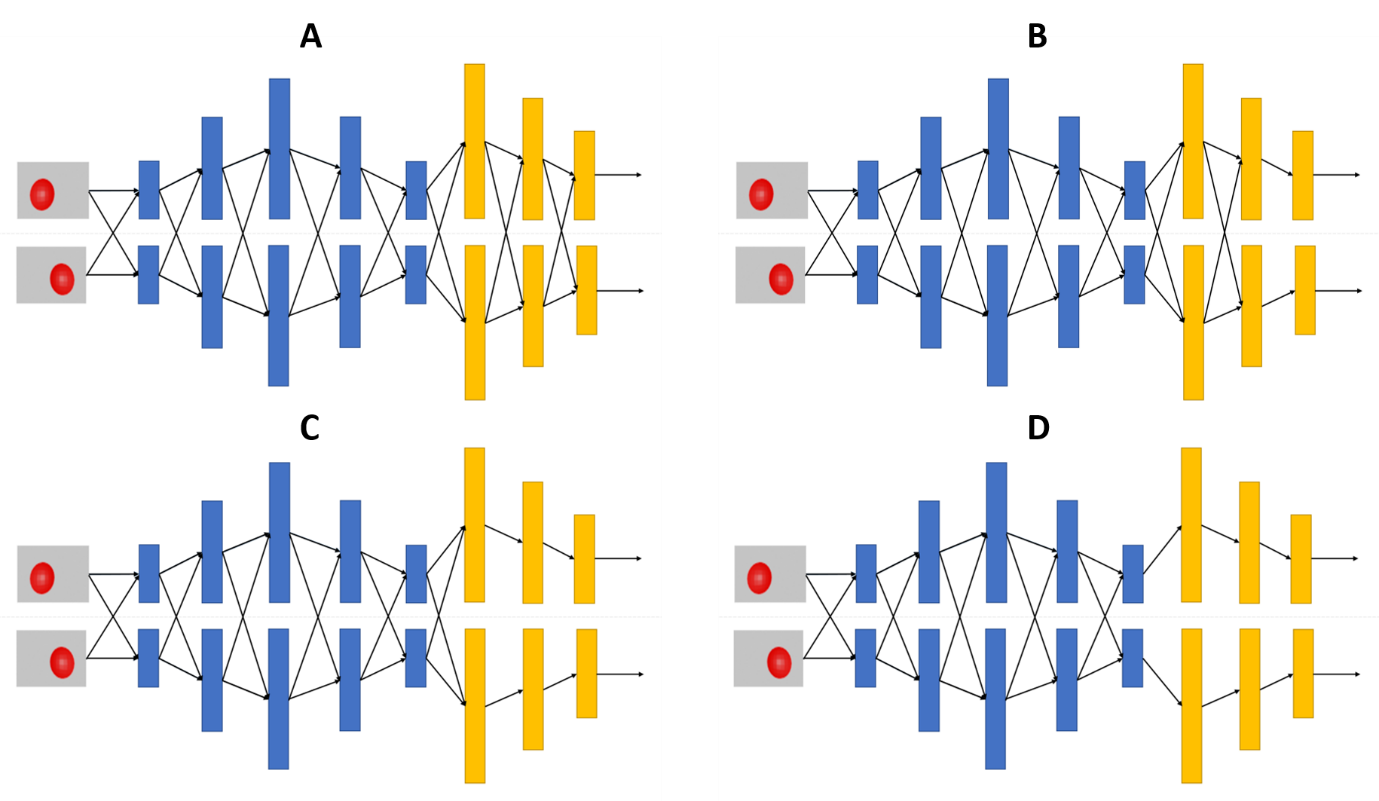


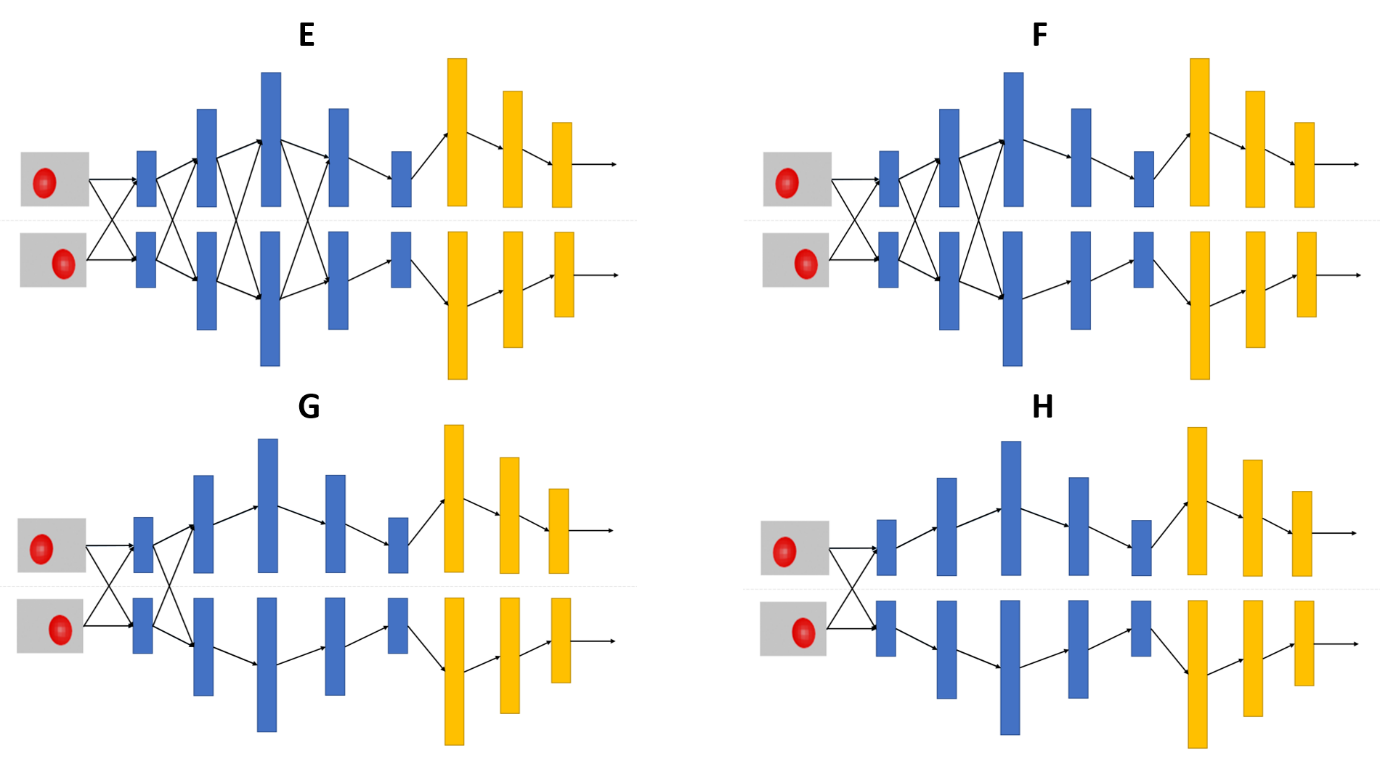


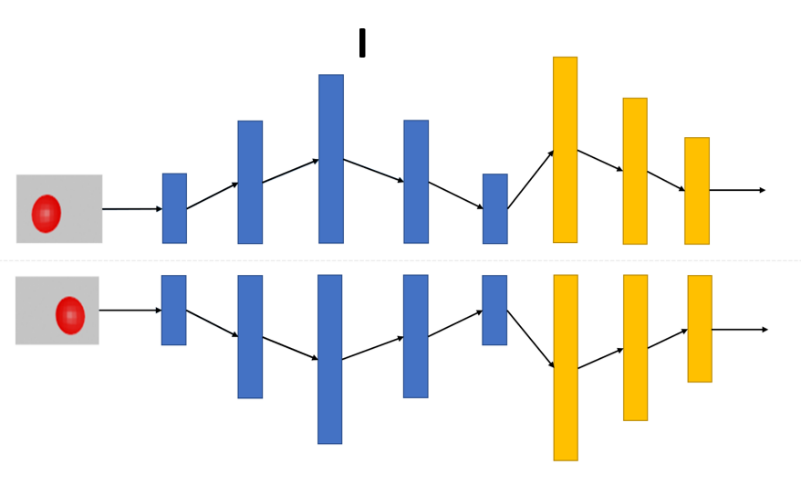


**Fig 4.** Removing cross-connections one layer at a time from output side to the input


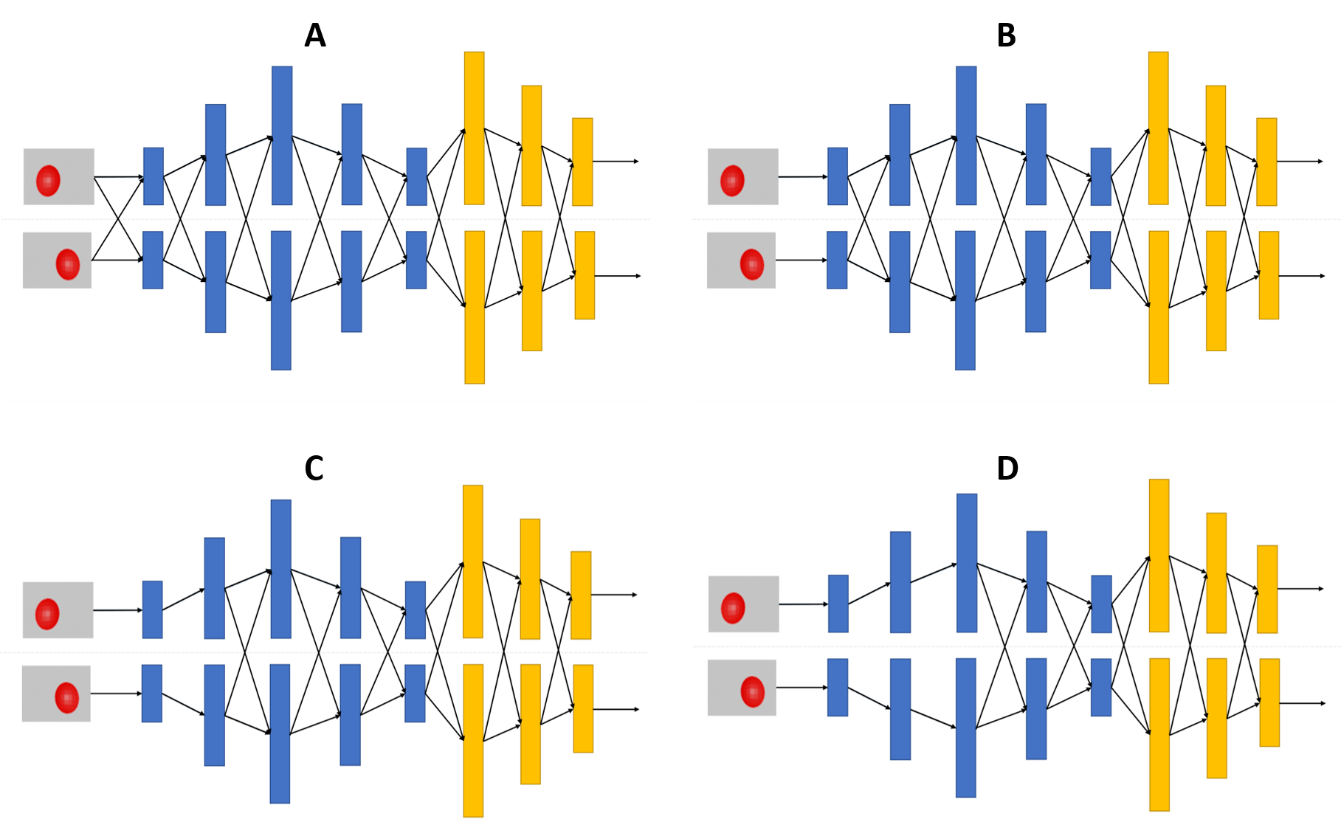


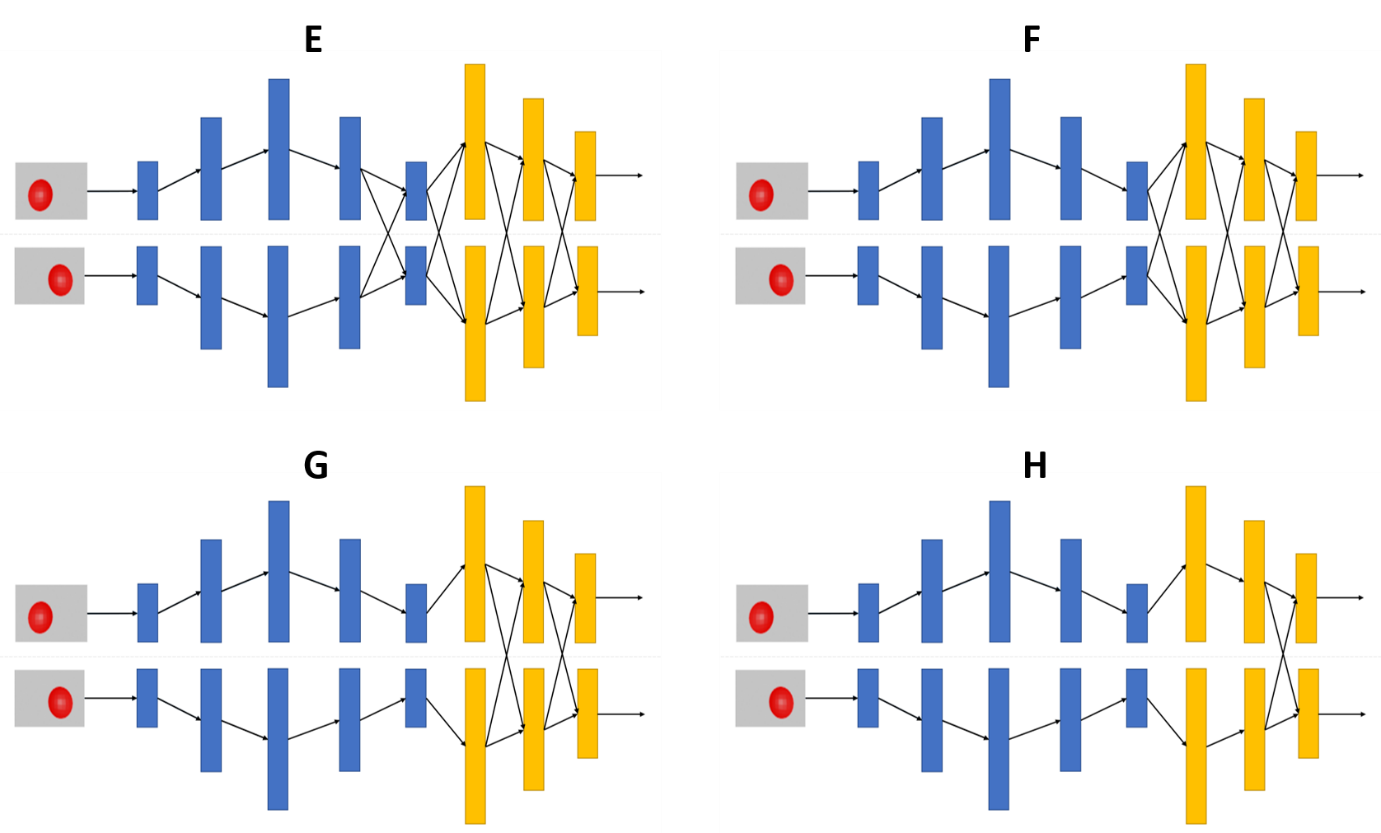


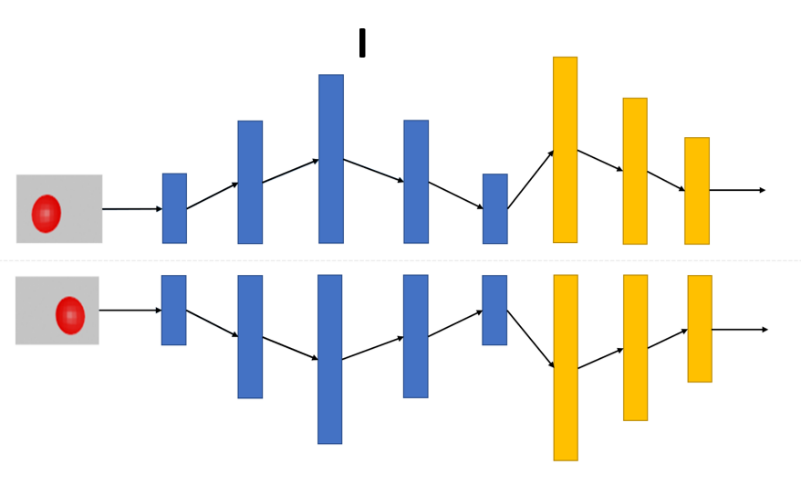


**Fig 5.** Removing cross-connections one layer at a time from input end to the output

# 4. Including cross-connections in just one layer –

# The cross-connections over the entire network were set to zero except for one layer in the network to generate the most minimal network in terms of the number of cross-connections (schematic shown in fig. 6). This procedure was done for all the layers in the network, and the performance was evaluated.


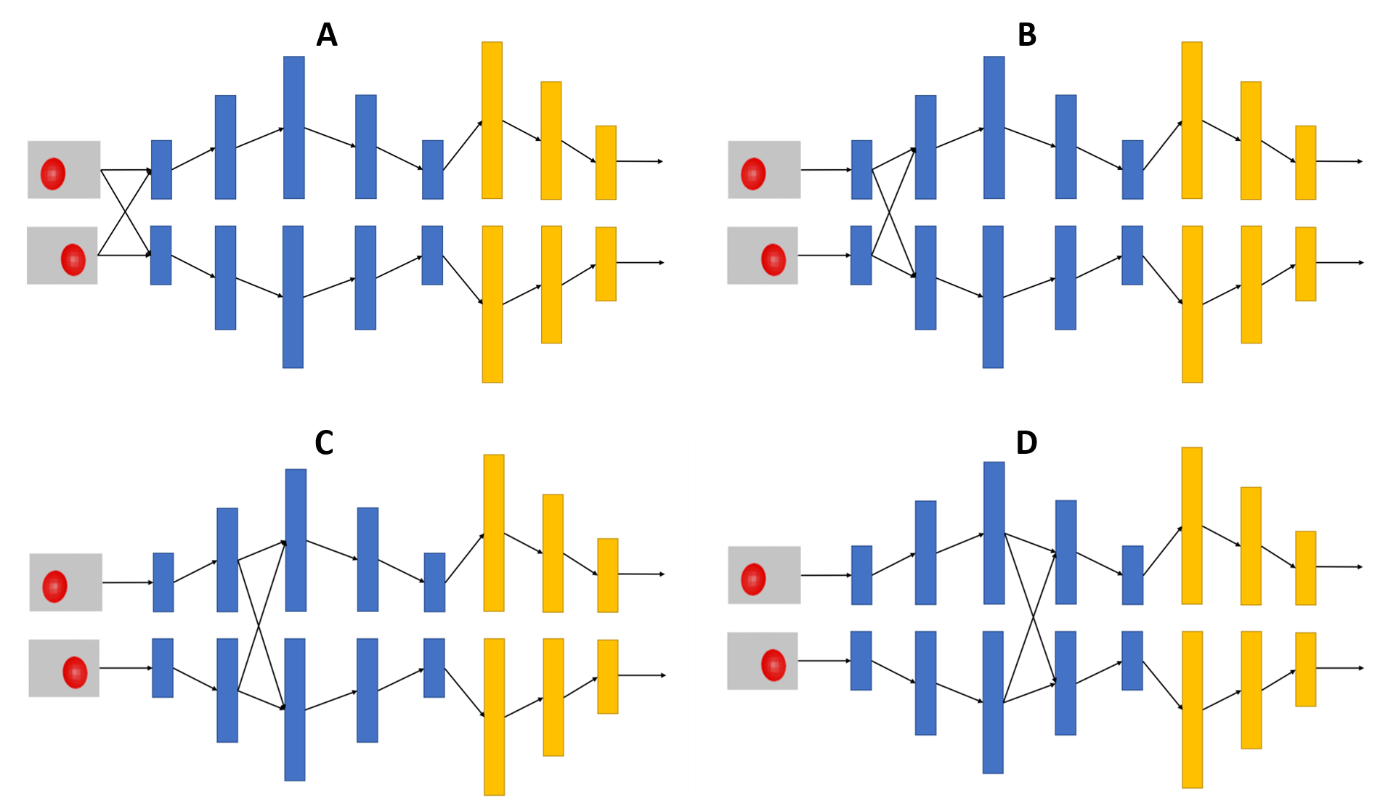


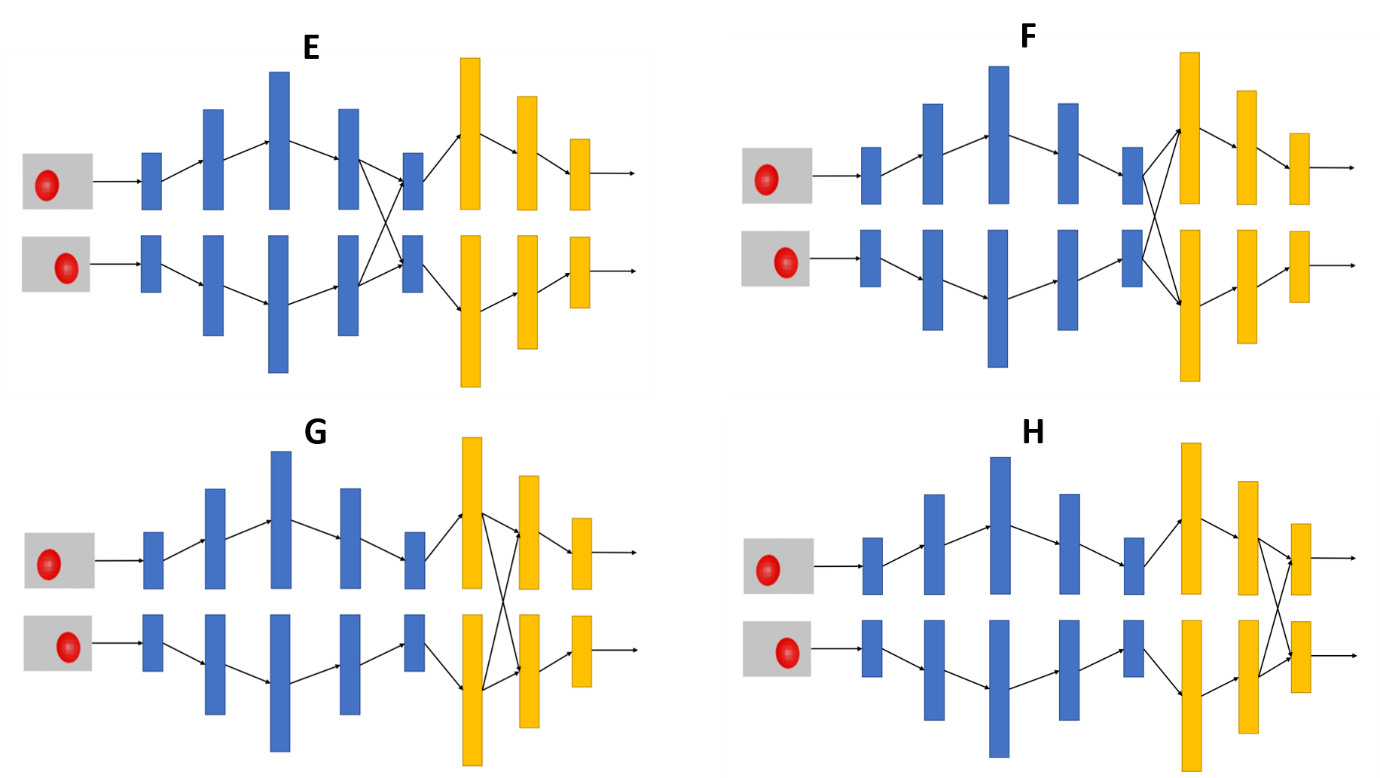


**Fig 6.** Having cross-connections only at one layer at a time

# Results

# Network performance was quantified with the help of Reaching Error (RE), defined as the Euclidean distance between the desired (target) and actual position of the arm,

$$RE= \left| X_{targ}-X_{arm} \right|$$

where, $X_{targ}$ is the 3D coordinates of the target

$X_{arm}$ is the 3D coordinates of the arm

# The network underwent several configuration changes, and the performance of the network was tested under each configuration.

# 1. Effect of removing cross-connections in the entire network –

# The network shows high error when there are no cross-connections in the network and low error when the cross-connections are included. This plot in fig. 7 shows that having cross-connections in the network is essential for high performance.

# 2. Effect of removing cross-connections in individual parts of the network

# Under this paradigm, the network was divided into two parts – visual and motor regions. The connections in one part of the network were varied while fixing the connections in the other part.

# 2.1. Varying cross-connections inside Visual Region

# In the graph shown in fig. 7, the blue solid and dashed lines indicate the case where cross-connections in the motor layers are fixed while varying the visual layer cross-connections. Both the curves show a downward trend as the cross-connections are included. However, the performance of this network does not vary much in comparison with the previous case of removing cross-connections from the entire network. This indicates that the performance of the network might not depend on the connections in the motor region as much.

# 2.2. Varying cross-connections in the motor region

# The solid and dashed red lines in fig. 7, indicate the network's performance when the cross-connections are fixed in the visual region, while varying the cross-connections in the motor region. When the network has cross-connections over the entire visual region, the performance does not vary regardless of the cross-connections over the motor region. Furthermore, this value is the lowest error that the different configurations achieve. However, when there are no cross-connections in the visual region, the error remains at a high value regardless of the connections at the motor region. Thus, this combined with what was observed when varying connections at the visual region, shows that the cross-connections in the visual region are more important than the cross-connections in the motor region for performing stereovision task.


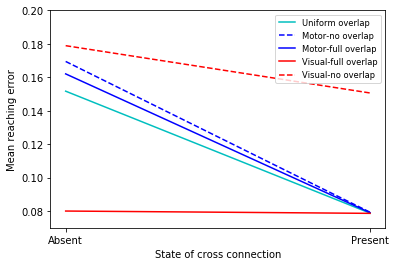


**Fig 7.** Performance of the network under the stereovision task under the different paradigms described above. [Dark Blue solid – Visual side cross-connections varied with Motor side having full cross-connections (Motor-full overlap); Dark Blue dashed – Visual side cross-connections varied with Motor side having no cross-connections (Motor-no overlap); Light Blue solid – Visual side and Motor side cross-connections varied (Uniform overlap); Red solid – Motor side cross-connections varied with Visual side having full cross-connections (Visual-full overlap); Red dashed – Motor side cross-connections varied with Visual side having no cross-connections (Visual-no overlap)]

# 3. The effect of removing cross-connections from one end of the network to the other

# 3.1. From Output (Motor) end to Input (Visual) end

# When the connections are removed from the output to the input, it may be noted that the connections in the motor region are removed first. The network shows a high error when there are no layers with cross-connections (number of layers where the cross-connections are cut = 8), whereas any other configuration, regardless of the actual number of layers that have cross-connections, shows a comparatively low error as seen by fig 8.

# 3.2. From Input (Visual) to Output (Motor) end

# In this case, the cross-connections are removed first from the visual end, and the connections from the motor region are removed at the end. Unlike the previous curve, here (red curve in fig 8.), the increase in error is more gradual. The network configurations containing cross-connections between two layers in the visual side (until number of layers where the cross-connections are cut = 4) show comparatively low error. But once the cross-connections in the visual side region are entirely removed (after number of layers where the cross-connections are cut = 5), the error starts increasing. These results, once again illustrate the importance of having cross-connections on the visual side.


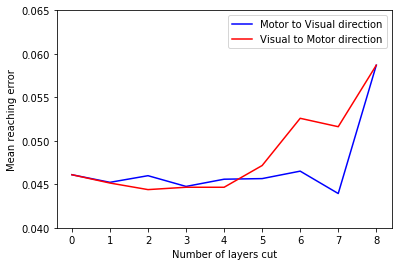


**Fig 8.** Performance of the network upon removing cross connections from one side to the other (network connections represented by figures 6 (Motor to Visual direction) and 7 (Visual to Motor direction))

# 4. The effect of having cross-connections at just one layer

# In this case, as suggested from the previous configurations, as the layer containing the cross-connections is placed farther and farther away from the input (closer to the motor side), the error increases as seen from fig. 9. The increase in error gets sharper as the layers move closer to the border of the visual region and the beginning of the motor region (layer 5).


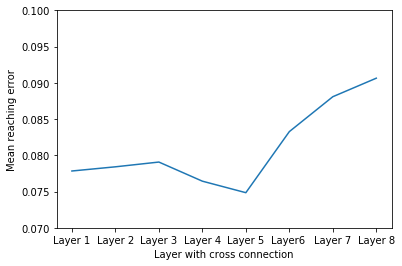


**Fig 9.** Performance of the network with cross connections at just one layer

# 5. Optimized network:

# The optimized network is shown in fig. 10 and it consists of five convolutional (visual) layers and three fully connected (motor) layers. Cross connections are provided in the visual region between 1st and 2nd convolutional layers and the motor side between 1st and 2nd fully connected layers. The HSPs are given to the network as input at the 3rd convolutional layer.

# The cross-connections present in the network can be can be thought of as the corpus callosum fibers in the brain as they serve a similar function of connecting the two halves of the network. Consolidating this analogy further, we present results showing the performance of the network when cross-connections are only present in one of the layers (fig. 9). Comparing the presence of cross-connections between two convolutional layers and between two fully connected layers, we can see that the network's performance is too low (high error) in the latter. This is similar to what is observed in patients with callosal sections. When the splenium of the corpus callosum (joining the visual cortical areas) are sectioned, while the rostrum and the body are intact (joins the motor and parietal areas)[1], patients report difficulty in identifying visual stimuli in the midline. The same is not observed when the stimuli are present in the periphery of the visual field or only visible to one eye[2]. In the dataset used here, the target positions are chosen such that they are visible to both eyes. No peripheral targets are used. This further strengthens the analogy made between the proposed network and the brain architecture subserving visuo-motor function.


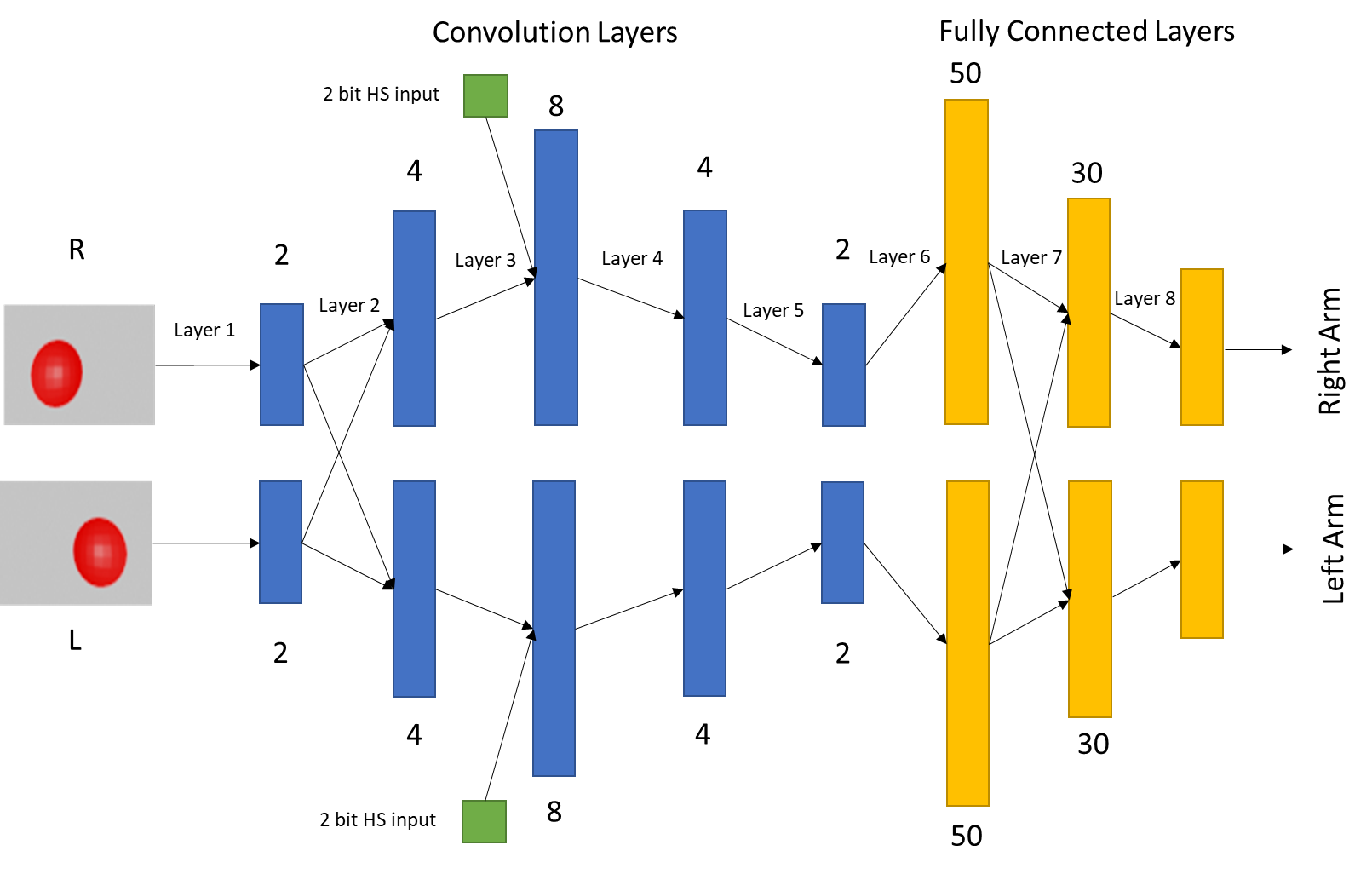


**Fig 10.** Block diagram illustrating architecture of the optimized Bilateral network.

# References

[1] Hofer S, Frahm J. Topography of the human corpus callosum revisited-Comprehensive fiber tractography using diffusion tensor magnetic resonance imaging. NeuroImage 2006;32:989–94. https://doi.org/10.1016/j.neuroimage.2006.05.044.

[2] Jeeves MA. Stereo perception in callosal agenesis and partial callosotomy. Neuropsychologia 1991;29:19–34. https://doi.org/10.1016/0028-3932(91)90091-L.
