# Supplementary material for: Interaction of network and rehabilitation therapy parameters in defining recovery after stroke in a Bilateral Neural Network
Source: J Neuroeng Rehabil. 2022 Dec 19;19:142. doi: 10.1186/s12984-022-01106-3 (PMC9762011; doi:10.1186/s12984-022-01106-3)
Supplement: Supplementary file 2 — Additional file 2. Statistical Analysis. [file 12984_2022_1106_MOESM2_ESM.docx]

**Additional File 2**

If the p-value under Levene test is greater than 0.005, then the groups have homogeneity of variance. These groups are then analyzed with one-way ANOVA or with welch-ANOVA correspondingly.

**Levene Test followed by ANOVA Test –**

| Stage of Recovery | CC Integrity | Lesion Size | Levene Test | ANOVA | Welch-ANOVA |
| --- | --- | --- | --- | --- | --- |
| ACUTE | 100 | 5 | 0.036984 | - | <0.001 |
|  |  | 10 | 0.302292 | <0.001 | - |
|  |  | 15 | 0.07818 | <0.001 | - |
|  |  | 20 | 0.038707 | - | <0.001 |
|  | 90 | 5 | 0.051636 | 0.6635 | - |
|  |  | 10 | 0.050539 | 0.907476 | - |
|  |  | 15 | 0.069644 | 0.342945 | - |
|  |  | 20 | 0.752002 | <0.001 | - |
|  | 70 | 5 | 0.229261 | 0.578817 | - |
|  |  | 10 | 0.120206 | 0.728491 | - |
|  |  | 15 | 0.073712 | 0.967651 | - |
|  |  | 20 | 0.248556 | <0.001 | - |
|  | 50 | 5 | 0.805113 | 0.041331 | - |
|  |  | 10 | 0.199197 | 0.133699 | - |
|  |  | 15 | 0.383122 | 0.650582 | - |
|  |  | 20 | 0.746289 | 0.001491 | - |
| CHRONIC | 100 | 5 | 0.01618 | - | 0.005 |
|  |  | 10 | 0.029798 | - | <0.001 |
|  |  | 15 | 0.968031 | <0.001 | - |
|  |  | 20 | 0.145663 | <0.001 | - |
|  | 90 | 5 | 0.393866 | 0.959842 | - |
|  |  | 10 | 0.235371 | 0.601618 | - |
|  |  | 15 | 0.870369 | 0.009354 | - |
|  |  | 20 | 0.935443 | <0.001 | - |
|  | 70 | 5 | 0.427109 | 0.423149 | - |
|  |  | 10 | 0.049834 | - | 0.005* |
|  |  | 15 | 0.584306 | 0.005289 | - |
|  |  | 20 | 0.165023 | <0.001 | - |
|  | 50 | 5 | 0.524011 | 0.915254 | - |
|  |  | 10 | 0.780396 | 0.643083 | - |
|  |  | 15 | 0.938567 | 0.120979 | - |
|  |  | 20 | 0.988132 | <0.001 | - |

**Table 1.** Table showing p-values obtained on Levene test followed by ANOVA with or without Welch correction. * - p-value = 0.366 on Dunn-Bonferroni post hoc test

**ACUTE STROKE –**

**Tukey HSD Test –**

Lesion size = 10 nodes, CC Integrity = 100%

| Group 1 | Group 2 | p-value |
| --- | --- | --- |
| 10SL | 10EL | 0.0368 |
| 10SL | 11SL | 0.9 |
| 10SL | 11EL | 0.0172 |
| 10SL | 10SG | 0.9 |
| 10SL | 10EG | 0.0074 |
| 10SL | 11SG | 0.9 |
| 10SL | 11EG | 0.1132 |
| 10EL | 11SL | 0.0303 |
| 10EL | 11EL | 0.9 |
| 10EL | 10SG | 0.0284 |
| 10EL | 10EG | 0.9 |
| 10EL | 11SG | 0.0151 |
| 10EL | 11EG | 0.9 |
| 11SL | 11EL | 0.0141 |
| 11SL | 10SG | 0.9 |
| 11SL | 10EG | 0.006 |
| 11SL | 11SG | 0.9 |
| 11SL | 11EG | 0.0953 |
| 11EL | 10SG | 0.0131 |
| 11EL | 10EG | 0.9 |
| 11EL | 11SG | 0.0068 |
| 11EL | 11EG | 0.9 |
| 10SG | 1OEG | 0.0055 |
| 10SG | 11SG | 0.9 |
| 10SG | 11EG | 0.0899 |
| 10EG | 11SG | 0.0028 |
| 10EG | 11EG | 0.9 |
| 11SG | 11EG | 0.0512 |

**Table 2.** p-values obtained on Tukey test after ANOVA. [10 – Constraint Induced Movement Therapy (CIMT), 11 – Bimanual Therapy (BMT), S – Stereotypic Therapy, E – Exploratory Therapy, L – Local Plasticity, G – Global Plasticity; Eg – 10SL – CIMT under Stereotypic environment with Local plasticity]

Lesion size = 15 nodes, CC Integrity = 100%

| Group 1 | Group 2 | p-value |
| --- | --- | --- |
| 10SL | 10EL | 0.001 |
| 10SL | 11SL | 0.9 |
| 10SL | 11EL | 0.001 |
| 10SL | 10SG | 0.8392 |
| 10SL | 10EG | 0.001 |
| 10SL | 11SG | 0.4893 |
| 10SL | 11EG | 0.001 |
| 10EL | 11SL | 0.001 |
| 10EL | 11EL | 0.9 |
| 10EL | 10SG | 0.004 |
| 10EL | 10EG | 0.2688 |
| 10EL | 11SG | 0.0204 |
| 10EL | 11EG | 0.9 |
| 11SL | 11EL | 0.001 |
| 11SL | 10SG | 0.7716 |
| 11SL | 10EG | 0.001 |
| 11SL | 11SG | 0.418 |
| 11SL | 11EG | 0.001 |
| 11EL | 10SG | 0.002 |
| 11EL | 10EG | 0.4019 |
| 11EL | 11SG | 0.0107 |
| 11EL | 11EG | 0.9 |
| 10SG | 1OEG | 0.001 |
| 10SG | 11SG | 0.9 |
| 10SG | 11EG | 0.001 |
| 10EG | 11SG | 0.001 |
| 10EG | 11EG | 0.5801 |
| 11SG | 11EG | 0.0047 |

**Table 3.** p-values obtained on Tukey test after ANOVA. [10 – Constraint Induced Movement Therapy (CIMT), 11 – Bimanual Therapy (BMT), S – Stereotypic Therapy, E – Exploratory Therapy, L – Local Plasticity, G – Global Plasticity; Eg – 10SL – CIMT under Stereotypic condition with Local plasticity]

A. Lesion size = 20 nodes, CC Integrity = 90%

| Group 1 | | Group 2 | p-value |
| --- | --- | --- | --- |
| 10L | 11L | | 0.9 |
| 10L | 10G | | 0.0108 |
| 10L | 11L | | 0.0113 |
| 11L | 10G | | 0.003 |
| 11L | 11G | | 0.0031 |
| 10G | 11G | | 0.9 |

B. Lesion size = 20 nodes, CC Integrity = 70%

| Group 1 | | Group 2 | p-value |
| --- | --- | --- | --- |
| 10L | 11L | | 0.9 |
| 10L | 10G | | 0.001 |
| 10L | 11L | | 0.0024 |
| 11L | 10G | | 0.001 |
| 11L | 11G | | 0.0024 |
| 10G | 11G | | 0.7634 |

C. Lesion size = 5 nodes, CC Integrity = 50%

| Group 1 | | Group 2 | p-value |
| --- | --- | --- | --- |
| 10L | 11L | | 0.4909 |
| 10L | 10G | | 0.0631 |
| 10L | 11L | | 0.0584 |
| 11L | 10G | | 0.5739 |
| 11L | 11G | | 0.5516 |
| 10G | 11G | | 0.9 |

D. Lesion size = 20 nodes, CC Integrity = 50%

| Group 1 | | Group 2 | p-value |
| --- | --- | --- | --- |
| 10L | 11L | | 0.9 |
| 10L | 10G | | 0.0085 |
| 10L | 11L | | 0.0457 |
| 11L | 10G | | 0.0048 |
| 11L | 11G | | 0.0264 |
| 10G | 11G | | 0.8169 |

**Table 4.** p-values obtained in Tukey test for different lesion sizes and CC integrity conditions. [10 – Constraint Induced Movement Therapy (CIMT), 11 – Bimanual Therapy (BMT), L – Local Plasticity, G – Global Plasticity; Eg – 10L – CIMT under Exploratory condition with Local plasticity]

**Dunn-Bonferroni Test –**

Lesion size = 5 nodes, CC Integrity = 100%

| Group 1 | Group 2 | p-value |
| --- | --- | --- |
| 11EL | 11SG | 1.000 |
| 11EL | 10EL | 1.000 |
| 11EL | 11EG | 1.000 |
| 11EL | 11SL | 0.359 |
| 11EL | 10SL | 0.263 |
| 11EL | 10SG | 0.162 |
| 11EL | 10EG | 0.126 |
| 11SG | 10EL | 1.000 |
| 11SG | 11EG | 1.000 |
| 11SG | 11SL | 0.387 |
| 11SG | 10SL | 0.285 |
| 11SG | 10SG | 0.176 |
| 11SG | 10EG | 0.137 |
| 10EL | 11EG | 1.000 |
| 10EL | 11SL | 0.913 |
| 10EL | 10SL | 0.693 |
| 10EL | 10SG | 0.450 |
| 10EL | 10EG | 0.359 |
| 11EG | 11SL | 1.000 |
| 11EG | 10SL | 1.000 |
| 11EG | 10SG | 1.000 |
| 11EG | 10EG | 0.976 |
| 11SL | 10SL | 1.000 |
| 11SL | 10SG | 1.000 |
| 11SL | 10EG | 1.000 |
| 10SL | 10SG | 1.000 |
| 10SL | 10EG | 1.000 |
| 10SG | 10EG | 1.000 |

**Table 5.** p-values obtained on Bonferroni test. [10 – Constraint Induced Movement Therapy (CIMT), 11 – Bimanual Therapy (BMT), S – Stereotypic Therapy, E – Exploratory Therapy, L – Local Plasticity, G – Global Plasticity; Eg – 10SL – CIMT under Stereotypic condition with Local plasticity]

Lesion size = 20 nodes, CC Integrity = 100%

| Group 1 | Group 2 | p-value |
| --- | --- | --- |
| 10EG | 11EG | 1.000 |
| 10EG | 11EL | 1.000 |
| 10EG | 10EL | 1.000 |
| 10EG | 11SG | 0.063 |
| 10EG | 10SG | 0.022 |
| 10EG | 10SL | 0.003 |
| 10EG | 11SL | 0.002 |
| 11EG | 11EL | 1.000 |
| 11EG | 10EL | 1.000 |
| 11EG | 11SG | 0.225 |
| 11EG | 10SG | 0.089 |
| 11EG | 10SL | 0.014 |
| 11EG | 11SL | 0.012 |
| 11EL | 10EL | 1.000 |
| 11EL | 11SG | 1.000 |
| 11EL | 10SG | 1.000 |
| 11EL | 10SL | 0.602 |
| 11EL | 11SL | 0.560 |
| 10EL | 11SG | 1.000 |
| 10EL | 10SG | 1.000 |
| 10EL | 10SL | 0.646 |
| 10EL | 11SL | 0.602 |
| 11SG | 10SG | 1.000 |
| 11SG | 10SL | 1.000 |
| 11SG | 11SL | 1.000 |
| 10SG | 10SL | 1.000 |
| 10SG | 11SL | 1.000 |
| 10SL | 11SL | 1.000 |

**Table 6.** p-values obtained on Bonferroni test. [10 – Constraint Induced Movement Therapy (CIMT), 11 – Bimanual Therapy (BMT), S – Stereotypic Therapy, E – Exploratory Therapy, L – Local Plasticity, G – Global Plasticity; Eg – 10SL – CIMT under Stereotypic condition with Local plasticity]

**CHRONIC STROKE –**

**Tukey HSD Test –**

Lesion size = 15 nodes, CC Integrity = 100%

| Group 1 | Group 2 | p-value |
| --- | --- | --- |
| 10SL | 10EL | 0.0017 |
| 10SL | 11SL | 0.9 |
| 10SL | 11EL | 0.011 |
| 10SL | 10SG | 0.1151 |
| 10SL | 10EG | 0.001 |
| 10SL | 11SG | 0.0797 |
| 10SL | 11EG | 0.001 |
| 10EL | 11SL | 0.001 |
| 10EL | 11EL | 0.9 |
| 10EL | 10SG | 0.6693 |
| 10EL | 10EG | 0.0187 |
| 10EL | 11SG | 0.7654 |
| 10EL | 11EG | 0.1513 |
| 11SL | 11EL | 0.001 |
| 11SL | 10SG | 0.0074 |
| 11SL | 10EG | 0.001 |
| 11SL | 11SG | 0.0047 |
| 11SL | 11EG | 0.001 |
| 11EL | 10SG | 0.9 |
| 11EL | 10EG | 0.0029 |
| 11EL | 11SG | 0.9 |
| 11EL | 11EG | 0.0319 |
| 10SG | 10EG | 0.001 |
| 10SG | 11SG | 0.9 |
| 10SG | 11EG | 0.0024 |
| 10EG | 11SG | 0.001 |
| 10EG | 11EG | 0.9 |
| 11SG | 11EG | 0.0039 |

**Table 7.** p-values obtained on Tukey test. [10 – Constraint Induced Movement Therapy (CIMT), 11 – Bimanual Therapy (BMT), S – Stereotypic Therapy, E – Exploratory Therapy, L – Local Plasticity, G – Global Plasticity; Eg – 10SL – CIMT under Stereotypic condition with Local plasticity]

Lesion size = 20 nodes, CC Integrity = 100%

| Group 1 | Group 2 | p-value |
| --- | --- | --- |
| 10SL | 10EL | 0.001 |
| 10SL | 11SL | 0.9 |
| 10SL | 11EL | 0.001 |
| 10SL | 10SG | 0.394 |
| 10SL | 10EG | 0.001 |
| 10SL | 11SG | 0.3948 |
| 10SL | 11EG | 0.001 |
| 10EL | 11SL | 0.001 |
| 10EL | 11EL | 0.9 |
| 10EL | 10SG | 0.063 |
| 10EL | 10EG | 0.001 |
| 10EL | 11SG | 0.0628 |
| 10EL | 11EG | 0.001 |
| 11SL | 11EL | 0.001 |
| 11SL | 10SG | 0.3012 |
| 11SL | 10EG | 0.001 |
| 11SL | 11SG | 0.3019 |
| 11SL | 11EG | 0.001 |
| 11EL | 10SG | 0.1749 |
| 11EL | 10EG | 0.001 |
| 11EL | 11SG | 0.1745 |
| 11EL | 11EG | 0.001 |
| 10SG | 10EG | 0.001 |
| 10SG | 11SG | 0.9 |
| 10SG | 11EG | 0.001 |
| 10EG | 11SG | 0.001 |
| 10EG | 11EG | 0.9 |
| 11SG | 11EG | 0.001 |

**Table 8.** p-values obtained on Tukey test. [10 – Constraint Induced Movement Therapy (CIMT), 11 – Bimanual Therapy (BMT), S – Stereotypic Therapy, E – Exploratory Therapy, L – Local Plasticity, G – Global Plasticity; Eg – 10SL – CIMT under Stereotypic condition with Local plasticity]

A. Lesion size = 15 nodes, CC Integrity = 90%

| Group 1 | | Group 2 | p-value |
| --- | --- | --- | --- |
| 10L | 11L | | 0.9 |
| 10L | 10G | | 0.0276 |
| 10L | 11L | | 0.0916 |
| 11L | 10G | | 0.033 |
| 11L | 11G | | 0.1078 |
| 10G | 11G | | 0.9 |

B. Lesion size = 20 nodes, CC Integrity = 90%

| Group 1 | | Group 2 | p-value |
| --- | --- | --- | --- |
| 10L | 11L | | 0.9 |
| 10L | 10G | | 0.0025 |
| 10L | 11L | | 0.0057 |
| 11L | 10G | | 0.0031 |
| 11L | 11G | | 0.007 |
| 10G | 11G | | 0.9 |

C. Lesion size = 15 nodes, CC Integrity = 70%

| Group 1 | | Group 2 | p-value |
| --- | --- | --- | --- |
| 10L | 11L | | 0.9 |
| 10L | 10G | | 0.0142 |
| 10L | 11L | | 0.0894 |
| 11L | 10G | | 0.017 |
| 11L | 11G | | 0.1053 |
| 10G | 11G | | 0.7601 |

D. Lesion size = 20 nodes, CC Integrity = 70%

| Group 1 | | Group 2 | p-value |
| --- | --- | --- | --- |
| 10L | 11L | | 0.9 |
| 10L | 10G | | 0.0046 |
| 10L | 11L | | 0.0087 |
| 11L | 10G | | 0.0044 |
| 11L | 11G | | 0.0084 |
| 10G | 11G | | 0.9 |

E. Lesion size = 20 nodes, CC Integrity = 50%

| Group 1 | | Group 2 | p-value |
| --- | --- | --- | --- |
| 10L | 11L | | 0.9 |
| 10L | 10G | | 0.0166 |
| 10L | 11L | | 0.005 |
| 11L | 10G | | 0.0178 |
| 11L | 11G | | 0.0053 |
| 10G | 11G | | 0.9 |

**Table 9.** p-values obtained on Tukey test. [10 – Constraint Induced Movement Therapy (CIMT), 11 – Bimanual Therapy (BMT), L – Local Plasticity, G – Global Plasticity; Eg – 10L – CIMT under Exploratory condition with Local plasticity]

**Dunn-Bonferroni Test –**

Lesion size = 5 nodes, CC Integrity = 100%

| Group 1 | Group 2 | p-value |
| --- | --- | --- |
| 10EG | 11EG | 1.000 |
| 10EG | 10EL | 1.000 |
| 10EG | 11EL | 1.000 |
| 10EG | 11SG | 0.797 |
| 10EG | 10SG | 0.418 |
| 10EG | 10SL | 0.063 |
| 10EG | 11SL | 0.025 |
| 11EG | 10EL | 1.000 |
| 11EG | 11EL | 1.000 |
| 11EG | 11SG | 1.000 |
| 11EG | 10SG | 1.000 |
| 11EG | 10SL | 0.225 |
| 11EG | 11SL | 0.098 |
| 10EL | 11EL | 1.000 |
| 10EL | 11SG | 1.000 |
| 10EL | 10SG | 1.000 |
| 10EL | 10SL | 1.000 |
| 10EL | 11SL | 1.000 |
| 11EL | 11SG | 1.000 |
| 11EL | 10SG | 1.000 |
| 11EL | 10SL | 1.000 |
| 11EL | 11SL | 1.000 |
| 11SG | 10SG | 1.000 |
| 11SG | 10SL | 1.000 |
| 11SG | 11SL | 1.000 |
| 10SG | 10SL | 1.000 |
| 10SG | 11SL | 1.000 |
| 10SL | 11SL | 1.000 |

**Table 10.** p-values obtained on Bonferroni test. [10 – Constraint Induced Movement Therapy (CIMT), 11 – Bimanual Therapy (BMT), L – Local Plasticity, G – Global Plasticity; Eg – 10L – CIMT under Exploratory condition with Local plasticity]

Lesion size = 10 nodes, CC Integrity = 100%

| Group 1 | Group 2 | p-value |
| --- | --- | --- |
| 10EG | 11EG | 1.000 |
| 10EG | 10EL | 1.000 |
| 10EG | 11EL | 0.913 |
| 10EG | 10SG | 0.521 |
| 10EG | 11SG | 0.418 |
| 10EG | 10SL | 0.008 |
| 10EG | 11SL | 0.005 |
| 11EG | 10EL | 1.000 |
| 11EG | 11EL | 1.000 |
| 11EG | 10SG | 1.000 |
| 11EG | 11SG | 0.853 |
| 11EG | 10SL | 0.022 |
| 11EG | 11SL | 0.014 |
| 10EL | 11EL | 1.000 |
| 10EL | 10SG | 1.000 |
| 10EL | 11SG | 1.000 |
| 10EL | 10SL | 1.000 |
| 10EL | 11SL | 1.000 |
| 11EL | 10SG | 1.000 |
| 11EL | 11SG | 1.000 |
| 11EL | 10SL | 1.000 |
| 11EL | 11SL | 1.000 |
| 10SG | 11SG | 1.000 |
| 10SG | 10SL | 1.000 |
| 10SG | 11SL | 1.000 |
| 11SG | 10SL | 1.000 |
| 11SG | 11SL | 1.000 |
| 10SL | 11SL | 1.000 |

**Table 11.** p-values obtained on Bonferroni test. [10 – Constraint Induced Movement Therapy (CIMT), 11 – Bimanual Therapy (BMT), S – Stereotypic Therapy, E – Exploratory Therapy, L – Local Plasticity, G – Global Plasticity; Eg – 10SL – CIMT under Stereotypic condition with Local plasticity]
